# Nrg1β Released in Remote Ischemic Preconditioning Improves Myocardial Perfusion and Decreases Ischemia/Reperfusion Injury via ErbB2-Mediated Rescue of Endothelial Nitric Oxide Synthase and Abrogation of Trx2 Autophagy

**DOI:** 10.1161/ATVBAHA.121.315957

**Published:** 2021-05-27

**Authors:** Venkatesh Kundumani-Sridharan, Jaganathan Subramani, Cade Owens, Kumuda C. Das

**Affiliations:** Department of Internal Medicine, Texas Tech University Health Sciences Center, Lubbock.

## Abstract

**Objective:**

Remote ischemic preconditioning (RIPC) is an intervention process where the application of multiple cycles of short ischemia/reperfusion (I/R) in a remote vascular bed provides protection against I/R injury. However, the identity of the specific RIPC factor and the mechanism by which RIPC alleviates I/R injury remains unclear. Here, we have investigated the identity and the mechanism by which the RIPC factor provides protection.

**Approach and ResultS:**

Using fluorescent in situ hybridization and immunofluorescence, we found that RIPC induces Nrg1β expression in the endothelial cells, which is secreted into the serum. Whereas, RIPC protected against myocardial apoptosis and infarction, treatment with neutralizing-Nrg1 antibodies abolished the protective effect of RIPC. Further, increased superoxide anion generated in RIPC is required for Nrg1 expression. Improved myocardial perfusion and nitric oxide production were achieved by RIPC as determined by contrast echocardiography and electron spin resonance. However, treatment with neutralizing-Nrg1β antibody abrogated these effects, suggesting Nrg1β is a RIPC factor. ErbB2 (Erb-B2 receptor tyrosine kinase 2) is not expressed in the adult murine cardiomyocytes, but expressed in the endothelial cells of heart which is degraded in I/R. RIPC-induced Nrg1β interacts with endothelial ErbB2 and thereby prevents its degradation. Mitochondrial Trx2 (thioredoxin) is degraded in I/R, but rescue of ErbB2 by Nrg1β prevents Trx-2 degradation that decreased myocardial apoptosis in I/R.

**Conclusions:**

Nrg1β is a RIPC factor that interacts with endothelial ErbB2 and prevents its degradation, which in turn prevents Trx2 degradation due to phosphorylation and inactivation of ATG5 (autophagy-related 5) by ErbB2. Nrg1β also restored loss of eNOS (endothelial nitric oxide synthase) function in I/R via its interaction with Src.

## Abstract 2

Supplemental Digital Content is available in the text.

**Highlights**

- Superoxide-induced Nrg1β (neuregulin 1β) released by the microvascular endothelial bed of the hindlimb acts as remote ischemic preconditioning factor and protects the myocardium from ischemia/reperfusion-induced injury.
- Loss of endothelial ErbB2 (Erb-B2 receptor tyrosine kinase 2 ) in ischemia/reperfusion induces dephosphorylation and activation of ATG5 (autophagy-related 5)-mediated Trx2 (thioredoxin 2) autophagy and apoptosis in ischemia/reperfusion.
- Remote ischemic preconditioning-dependent protection of endothelial ErbB2 inactivates ATG5 due to its phosphorylation, which prevents Trx2 autophagy.
- Endothelial-specific deletion of ErbB2 in mice results in loss of remote ischemic preconditioning-mediated protection of cardiac perfusion.
- Endothelial ErbB2 dimerizes with ErbB4, recruits Src and mediates Nrg1-dependent eNOS (endothelial nitric oxide synthase) activation resulting restoration of nitric oxide production, vasorelaxation and improved myocardial perfusion, as adult mice do not express ErbB2 in cardiomyocytes.

**See accompanying editorial on page 2315**

Ischemic heart disease is a leading cause of cardiovascular morbidity and mortality world wide.^1,2^ ST-segment–elevation myocardial infarction (MI) is a major emergency manifestation of ischemic heart disease. Reperfusion using percutaneous coronary intervention is the treatment of choice for reducing the size of MI’s, preserving left ventricular systolic function, and preventing of the onset of heart failure.^2^ In patients with ischemic heart disease and severe multi-vessel coronary disease, the heart is more commonly revascularized using coronary artery bypass graft surgery.^3^ Importantly, myocardial injury and cardiomyocyte death after coronary artery bypass graft surgery are caused by acute ischemia/reperfusion (I/R) similar to that seen in revascularization after ST-segment–elevation myocardial infarction.^2^ For both of these patient groups, ischemic preconditioning (IPC) can protect the heart from the detrimental effects of I/R; this preconditioning is a process where short periods of I/R protect against injury arising from more acute I/R. It has also been established that short periods of I/R in a remote vascular bed or organ can provide protection against I/R, resulting in decreased MI.^4^ For example, remote ischemic preconditioning (RIPC) before primary percutaneous coronary intervention treatment reduced size of infarcts in randomized trials.^2^ Additionally, consistent beneficial outcomes of RIPC in cardiac surgery and coronary angioplasty have been established^2^ and supported by recent clinical trials.^5^ RIPC is clinically effective and noninvasive; for example, in several clinical trials, therapeutic ischemia-reperfusion of the forearm has proven to protect the heart against I/R-induced MI.^1,2,6^ However, the specific protective factor (or even whether it is humoral, neural, or other) released by RIPC has not been identified.^2^ Further, the mechanism by which this factor confers protection to the heart during I/R remains poorly understood.^4,6,7^ Such understanding is clinically important if RIPC were to be optimized as an adjuvant in the management of MI and other ischemic diseases. It has been established that localized RIPC releases neural and humoral factors that provide beneficial effects for a distant organ. For example, blood from rabbits subjected to IPC can decrease the size of MI’s formed in response to IPC of naive rabbits, demonstrating that RIPC releases cardioprotective factors to the circulation.^8^ However, the mechanisms behind these effects are unclear, and the identity of cardioprotective factor released in response to RIPC has not been established.^5,7^

Nrgs (neuregulins) are polypeptides belonging to a subclass of epidermal growth factors that are secreted by endothelial cells.^9–11^ Nrgs play an indispensable role in cardiac development, and their deficiency causes mid-embryonic lethality due to cardiac trabeculae dysfunction.^12^ Based on its role in cardiac protection^13^ and repair,^14^ neuregulin has been tested as a therapeutic agent in clinical trials for the treatment of chronic heart conditions.^15,16^ Although the interaction of endothelial-secreted Nrg1β with cardiomyocyte ErbB (Erb-B2 receptor tyrosine kinase) receptors is well established in rat or other animal models,^17–19^ the interaction of adult murine cardiomyocyte ErbB2 with Nrg1β remains unknown. Studies have shown that murine ErbB2 expression is decreased to almost 99% in the heart at postnatal day 7 and further decline at P28.^20^ Besides, another study has shown a severe decrease (95%) in ErbB2 in the hearts of mice at 12 weeks of age.^21^ For example, deleting ErbB2 in knockout mice or treatment with ErbB2 inhibitor herceptin (trastuzumab) causes heart failure.^3,22^ Nrg1 signaling in the adult heart is mediated by ErbB2/ErbB4 heterodimers,^23^ which modulate cardiomyocyte contractile functions.^22^

Although ErbB2 is intensely studied in the context of cancer biology and treatment,^24,25^ studies involving its role in MI are limited. Besides, its role in RIPC has never been studied. Recent studies have shown that overexpression of ErbB2 in a cardiac-specific manner in 2-day old mice (P2) induces the expression of antioxidant genes, such as mitochondrial GPx1 (glutathione peroxidase 1) and catalase in the heart.^26^ However, the effect of RIPC on antioxidant response and its role in the protection of myocardial I/R injury is unknown. Mitochondrial Trx2 (thioredoxin-2) knockout mice die in utero due to massive apoptosis in the heart,^27,28^ suggesting a critical role of Trx2 in the protection of the heart. Further, Trx2 has been shown to inhibit mitochondrial reactive oxygen species (ROS) generation and ASK1 (apoptosis signal-regulating kinase 1) to maintain cardiac function.^29^ However, the role of Trx2 in I/R or RIPC-mediated protection against MI is unknown. In addition, regulation of Trx2 by ErbB2-mediated phosphorylated ATG5 (autophagy-related 5) has not been reported. Further, the antioxidant-inducing role of ErbB2 in I/R has never been reported. Additionally, how the protection of endothelial ErbB2 by RIPC decreases MI in I/R remains unclear.

## Materials and Methods

Data will be available from the corresponding author upon resonable request.

### Reagents

Recombinant human Nrg1β1 active domain (catalog No. 396-HB) and neutralizing anti-Nrg1 antibody (catalog No. AF-396-NA) were bought from R&D Systems. Anti-eNOS (endothelial nitric oxide synthase) antibody (catalog No. 610296) was obtained from BD Biosciences. Anti-Nrg1 (to detect full-length Nrg1, catalog No. 2573), anti-ErbB2 (catalog No. 4290), anti-phospho-eNOS (Ser1177; catalog No. 9571), anti-phospho-Src (Tyr416; catalog No. 2101), anti-Ambra1 (catalog No. 24907), and anti-BCLN1 (catalog No. 3495) antibodies were purchased from Cell Signaling Technology (Danvers, MA). Anti-Nrg1 antibody used for immunofluorescence (catalog No. SC-28916), anti-ErbB4 antibody (catalog No. SC-283), anti-VE-cadherin antibody (catalog No. SC-6458), anti-Trx2 antibody (catalog No. SC-50336), and MGD sodium salt monohydrate (catalog No. SC-221941A) were obtained from Santa Cruz Biotechnology (Dallas, TX). 1-Hydroxy-3-methoxycarbonyl-2,2,5,5-tetramethylpyrrolidine hydrochloride (CMH) was obtained from Enzo Lifesciences (catalog No. ALX-430-117-M250). PP1 (catalog No. 529579) was procured from EMD Millipore Chemicals (CA). Antiheregulin antibody (catalog No. RB-276-P0), anti-ErbB4 neutralizing antibody (catalog No. MA5-13016), and anti-ErbB4 antibody for proximity ligation assay (PLA; catalog No. MA1-861) were purchased from Pierce Biotechnology (Rockford, IL). Anti-ErbB2 antibody (catalog No. GTx117479) used for PLA was bought from GeneTex. Anti-Src antibody (catalog No. 05-184) and anti-phospho-Tyr antibody (catalog No. 05-321) were purchased from Millipore. Anti-CD31 (catalog No. ab9498), anti-ATG5 (catalog No. ab108327), and anti-Tom20 antibodies (catalog No. ab56783) antibodies were bought from Abcam. Neutralizing anti-ErbB2 antibody trastuzumab, which is also known as herceptin (catalog No. Ab00103-10.0) was obtained from Absolute Antibody Ltd via Labscoop LLC. Mouse Nrg1 ELISA kit (catalog No. EKU 06197) was purchased from Biomatik USA. Annexin-V-paramagentic iron microbeads were purchased from Miltenyi Biotec GmbH, Germany (catalog No. 130-090-201). Annexin V-FITC used in flowcytometry was bought from BD Bioscience (catalog No. 556547). Vevo MicroMarker nontargeted microbubbles (catalog No. VS-11913) used in nonlinear echocontrast imaging was obtained from Visualsonics. NOS activity assay kit (catalog No. 781001) was procured from Cayman Chemical Company. ProLong Gold Antifade Mountant (catalog No. P36930), Alexa Fluor 647 conjugated isolectin GS-IB4 (catalog No. I32450), and Alexa Fluor 488 or Alexa Fluor 568 conjugated secondary antibodies were obtained from Life Technologies. Human nontarget (NT) siRNA (catalog No. D-001810-10), human Nrg1 siRNA (catalog No. L-004608-02-0010), human ErbB2 siRNA (catalog No. L-003126-00-0010), human ErbB4 siRNA (catalog No. L-003128-00-0010), human Trx2 siRNA (catalog No. L-017448-01-0020), human beclin 1 siRNA (catalog No. L-010552-00-0010), and human AMBRA1 siRNA (catalog No. L-029987-01-0010) were purchased from Dharmacon. Construction of Ad-dnSrc and production of antiphospho-eNOS (Tyr 83) antibodies were described previously.^30,31^ pcDNA3-ErbB2 plasmid^32^ was a gift from Mein-Chie Hung and was obtained through Addgene (plasmid No. 16257).

### Cell Culture and Hypoxia-Reoxygenation

Human coronary artery endothelial cells (HCAEC) and human endocardial microvascular endothelial cells (HMVEC) were obtained from Lonza (Walkersville, MD) and were grown in EGM-2 supplemented with MV Bullet Kit. To expose HCAECs to hypoxia-reoxygenation (H/R), HCAECs were grown to confluence and then incubated with deoxygenated (flushed with 95% N_2_/5% CO_2_)-EGM-2 in Billups-Rothenberg modular chambers at 37 °C in 95% N_2_/5% CO_2_ gas mixture for an indicated period followed by reoxygenation in 5% CO_2_/95% air. To obtain preconditioned medium (PM), HMVECs were incubated with deoxygenated-EGM-2 without growth supplements for 1 hour at 37 °C in Billups-Rothenberg modular chambers flushed with a 95% N_2_/5% CO_2_ gas mixture. After 1 hour of hypoxia, the cells were reoxygenated in normoxic conditions. At this point, medium from HMVECs was collected and used as PM to treat HCAECs. Cardiomyocytes and endothelial cells from adult mouse hearts were isolated by Liberase TH (Roche Applied Science) and collagenase II digestion, respectively.^33^

### Transfections and Transductions

HCAEC were transfected with NT or specific siRNA molecules at a final concentration of 100 nmol/L or indicated concentration using Lipofectamine RNAiMAX transfection reagent (Life Technologies, Grand Island, NY) according to the manufacturer’s instructions. When adenoviral vectors were used to downregulate the function of Src, cells were transduced with adenovirus harboring either GFP (green fluorescent protein) or dnSrc at 40 multiplicities of infection overnight in EGM-2 supplemented with MV Bullet Kit. After 48 hours, transfections or transductions, cells were subjected to appropriate treatments.

### Animals

C57BL/6 (substrain 6NCrl) adult male (10–12 weeks) mice were purchased from Charles River Laboratory and used in this study. ErbB2^fl/fl^ embryos^34^ were procured from the European Mouse Mutant Achieve rederived in Charles River Laboratory. Only adult male mice were used in this study. Sex-related differences in cardiac sensitivity to ischemia-reperfusion injury and reduced risk in female sex are well documented. To eliminate the heterogeneity affecting the study, we did not include female mice. However, we plan to conduct further studies in females in RIPC protection. VE Cad CreER^T2^ strain^35^ was a kind gift from Luisa Iruela-Arispe. To generate a mouse model of endothelial-specific inducible deletion of ErbB2, we cross-bred ErbB2^fl/fl^ with VECad-Cre-ER^T2^ mice. To induce deletion of ErbB2 in endothelial cells, ErbB2^fl/fl^:VECad-Cre-ER^T2^ mice were injected with three doses of tamoxifen (75 mg/kg) in 24 hours intervals. All animal procedures were approved by the Institutional Animal Care and Use Committee of the TTUHSC and were consistent with the Guide for the Care and Use of Laboratory Animals published by the National Institute of Health.

### Remote IPC

To induce RIPC, mice were anesthetized with ketamine, 100 mg/kg (Ketathesia) and xylazine, 10 mg/kg (Anased, akorn animal Health). To expose femoral vessels, a longitudinal incision was made in the left groin region of the mouse, and the femoral vein and femoral artery (FA) were gently separated from the left femoral nerve. To induce ischemia, the FA and vein were transiently occluded at the downstream proximal region to the origin of the profunda femoris artery for 5 minutes using vascular clamps. Hindlimb reperfusion for the following 5 minutes was performed by release the FA and vein from the vascular clamp. Half-life of circulating Nrg1 is reported to be ≈30 minutes.^36–38^ Therefore, following 30 minutes of the last I/R cycle, mice were subjected to myocardial I/R. Mice were injected with 2 doses of Nrg1 neutralizing antibody (150 µg/kg) via tail vein at 12 hours (first dose) and 30 minutes (second dose) before RIPC as required.

### Myocardial Ischemia-Reperfusion

Mice (8–12 weeks) were anesthetized with an intraperitoneal injection of ketamine,100 mg/kg (Ketathesia) and xylazine, 10 mg/kg (Anased, akorn animal Health) and intubated for assisted respiration using a small animal ventilator (Harvard Apparatus, Natick, MA). Under a stereomicroscope, the chest cavity was opened with lateral cut along the left side of the sternum by cutting through the ribs to approximately mid sternum. The pericardium was gently dissected to visualize the coronary artery. To initiate myocardial ischemia, the left anterior descending artery was temporarily closed by a slip knot using an 8-0 monofilament polypropylene suture at a point two-thirds of the way between its origin near the pulmonary conus and the cardiac apex (1–2.5 mm from the tip of left atrium). A 1 mm section of PE-10 tubing was placed on top of left anterior descending artery while occluding to aid in the release of knot to start reperfusion. Sham mice underwent the same procedure without the slipknot tied. After the reperfusion, euthanasia was performed by dissecting the heart out and washed with physiological saline, while the animal was under anesthesia.

### Isolation of Left Coronary Artery Segments

Left coronary artery was isolated as described by us previously.^39^ Briefly, mice were euthanized by an overdose of ketamine/xylazine and the heart was removed and placed in cold Krebs-Ringer buffer with the following composition (in mmol/L): 118.5 NaCl, 4.7 KCl, 2.5 CaCl_2_, 1.2 MgSO_4_, 1.2 KH_2_PO_4_, 25.0 NaHCO_3_, and 5.5 D-glucose. The left coronary artery was carefully freed of cardiac tissue, and a segment (2–3 mm) was dissected for the eNOS activity assay.

### Myocardial Contrast Echocardiography

Regional hypoperfusion in mouse left ventricle walls was analyzed and quantitated by nonlinear contrast echocardiography. Vevo3100 high-resolution echo imaging system (Visual Sonics) was used to collect high-frequency ultrasound echo imaging from mouse heart. Mouse heart was scanned from long-axis view using 18 MHz high-resolution microscan transducer (MX250) with 10% maximal power, and contrast mode images were collected with 30 dB gain. After subjecting mice to sham or I/R surgery, 50 µL bolus dose of nontargeted contrast agent (2.0×105 microbubbles/µL) was administered via tail or femoral vein while collecting contrast echo imaging. Images were analyzed by Vevo CQ software for the rate of appearance and intensity of contrast (microbubbles) in the anterior wall region of left ventricles downstream to the left anterior descending artery. The appearance of the contrast agent in the left ventricle cavity was set as time zero for all calculations to quantitate perfusion of myocardial tissue. The ratio between area under the contrast intensity curve and time to peak was calculated as the local cardiac tissue perfusion index.

### Tissue Apoptosis Using EPR Spectrometry

To quantify total apoptosis in infarcted tissue of mouse heart, we employed a modified electron spin resonance (EPR)-based assay method as reported in our previous publications^40,41^ using annexin-V conjugated with paramagnetic iron from Miltenyi Biotec GmbH, Germany (catalog No. 130-090-201). After sham or IR surgery, the heart was quickly isolated from mice, cannulated via aortic arch, and perfused with ice-cold saline followed by 1× annexin-V binding buffer provided by the manufacturer. Following complete removal of circulating blood, heart was perfused with 250 µL of annexin-V microbead suspension and incubated at 2 to 4 °C for 20 minutes. At the end of the incubation period, the heart was perfused with ice-cold 1× annexin-V binding buffer, and the entire infarcted tissue was dissected out. Total annexin-V bound to infarcted tissue was quantified by measuring conjugated iron spins using Bruker EMX Nano spectrometer at room temperature. EPR spectra were acquired under following scan conditions: microwave frequency, 9.63 GHz; power, 0.32 mW; attenuation 25 dB; modulation frequency, 100 kHz; modulation amplitude, 4.00 G; sweep time, 60 s; time constant, 20.48 s; receiver gain, 40 dB; and magnetic field, 1610-4610 G. The figures presented show the first order derivatives from electron spin resonance data under fixed range magnetic field. From the spectra, absolute spin counts were obtained using internal spin calibration using Bruker Xenon data processing program using quantitative EPR module of Bruker Xenon Nano 1.3 software.

### TTC Staining

Myocardial infarct size was determined by triphenyltetrazolium chloride (TTC) staining as described previously.^42^ Briefly, after reperfusion, animals were euthanized and aorta was cannulated, perfused with saline to remove blood. 0.25 mL of 1.5% Evans blue was perfused after religating the coronary artery to demarcate remote myocardium (blue) and area at risk (AAR). Then the heart was sectioned at 1.0 mm thickness, and the heart sections were stained with 1.0% TTC for 15 minutes at 37 °C. TTC stained heart sections were fixed with paraformaldehyde and photographed using Nikon D5200 camera using Nikon AF-S DX NIKKOR 18 to 55 mm lens at f/6.3, 1/160s, ISO200. TTC stained and unstained area (infarct) at AAR was quantified using Adobe Photoshop.

### Western Blotting and Immunoprecipitation

After appropriate treatments and rinsing with cold PBS, endothelial cells were lysed in 500 µL of lysis buffer (20 mmol/L Tris-HCl, pH 7.4, 150 mmol/L NaCl, 1% Nonidet P-40, 0.5% sodium deoxycholate, 0.1% SDS, 2 mmol/L phenylmethylsulfonyl fluoride, 100 µg/mL aprotinin, 1 µg/mL leupeptin, and 1 mmol/L sodium orthovanadate) and scraped into 1.5 mL Eppendorf tubes. After standing on ice for 20 minutes, the cell and tissue extracts were cleared by centrifugation. Protein in the cell lysates was quantified using BCA assay kit (Pierce, Rockford, IL). Western analysis was performed using the Bio-Rad mini protean system. Equal amounts of protein were resolved on a 6% to 15% SDS-PAGE. Following electrophoresis, the protein was transferred to a nitrocellulose (Hybond-ECL, GE Healthcare) or polyvinylidene difluoride membrane, immunoblotted with respective primary antibodies, and visualized by the enhanced chemiluminescence system (GE Healthcare) using appropriate secondary antibodies conjugated with horseradish peroxidase IgG. Some of the images were acquired with G:BOX Chemi XL1.4 (Syngene, Frederick, MD) using West Femto reagent from Pierce. In case of immunoprecipitation, an equal amount of protein from control and each treatment was incubated with appropriate antibody overnight at 4 °C followed by protein A conjugated Sepharose CL4B beads for 45 minutes at 25 °C. The beads were collected by centrifugation, washed in lysis buffer, boiled in Laemmeli sample buffer for 5 minutes, the released proteins were resolved on SDS-PAGE and Western blotting was performed.

### ELISA

To collect serum from mice, mice were first subjected to RIPC. Thirty minutes after the last cycle of hindlimb I/R cycle, mice were euthanized and blood was collected from left ventricle into a serum separator tube. Blood was allowed to clot for 2 hours at room temperature, and serum was separated by centrifugation at 1000*g* for 20 minutes. Nrg1 in serum was determined by ELISA kit (EKU06197, Biomatik, Wilmington, DE) following supplier’s protocol.

### Reverse Transcription Polymerase Chain Reaction

After appropriate treatments, total cellular RNA was isolated from HMVEC using Trizol reagent as per manufacturer’s instructions (Life Technologies). Reverse transcription was performed with the High-Capacity cDNA Reverse Transcription Kit (Applied Biosystems) following the supplier’s protocol. The cDNA was used as a template for polymerase chain reaction (PCR) using specific primers. The primers used are as follows: human Nrg1, 5′-GACCTCTACTTCTCGTGACA-3′ (forward) and 5′- TCCAATCTGTTAGCAATGTG -3′ (reverse); human β-actin, 5′- TCTAGGCACCAAGGTGTG-3′ (forward), and 5′-TCATGAGGTAGTCCGTCAGG-3’ (reverse). The PCR performed on T-100 thermal cycler (Bio-Rad, Hercules, CA). The amplified RT-PCR products were separated on 1.6% (w/v) agarose gels and stained with ethidium bromide, pictures were captured using G:BOX Chemi XL1.4 (Syngene, Frederick, MD).

### Immunofluorescence Staining

After 30 minutes of the last I/R cycle of RIPC, mice were euthanized and the hindlimb was perfused with saline via FA and fixed with z-fix fixative. Gastrocnemius muscle along with surrounding tissues were carefully dissected out and embedded with paraffin. Paraffin-embedded tissue was sliced at a thickness of 5 µm using a microtome and mounted onto a microscope slide. Slide mounted tissue sections were deparaffinized, permeabilized, and subjected to antigen unmasking by incubating in 10 mmol/L sodium citrate pH 6.0 at 60 °C for 2 hours. Thereafter, the tissue sections were probed with anti-Nrg1 and anti-CD31 antibodies followed by Alexa Fluor 488 or Alexa Fluor 568-conjugated secondary antibodies and Alexa Fluor 594-conjugated isolectin GS-IB4. Fluorescence images were obtained via 20×/0.8 NA objective using Zeiss Axio Imager Z2 upright fluorescent microscope. To quantitate Nrg1 fluorescent signals, a capillary mask was generated using fluorescence image obtained from Alexa Fluor 594 channel and superimposed on Nrg1 image and the mean fluorescence density was quantitated using Adobe Photoshop.

### Flow Cytometry

HCAECs cells undergoing apoptosis were determined by annexin-V binding and detection by fluorescence-activated cell sorting analysis. After appropriate treatments, HCAECs were trypsinized using TrypLE reagent. The collected cells were washed and incubated with 1× annexin V binding buffer containing Annexin V-FITC (catalog No. 556547, BD Bioscience) as per manufacturer instructions. After incubation, the cells were washed with 1× binding buffer and subjected to fluorescence-activated cell sorting analysis using Attune NxT Flow Cytometer, and the results were analyzed using FlowJo software.

### In Situ PLA

In situ PLA was performed as described.^43^ HCAEC were seeded onto glass coverslips and allowed to grow to 90% confluence. After appropriate treatments, the cells were fixed with 3% paraformaldehyde prepared in PBS for 10 minutes at 37 °C. In case of cardiac tissue specimens, deparaffinized heart sections were permeabilized with 0.1% Triton X-100 for 10 minutes at room temperature, blocked with 5% donkey serum and 3% BSA in PBS for 1 hour at room temperature, and incubated with primary antibodies in 50% Da Vinci Green antibody diluent (Abcam, Cambridge, MA). PLA was performed following the supplier’s instruction using Duolink Anti-Rabbit PLUS and anti-mouse MINUS PLA probes and Duolink green detection reagent (Duolink, Sigma). To mark the border of the cells, they were stained with goat anti-VE-cadherin antibodies followed by Alexa Fluor 594-conjugated secondary antibodies. Thirty to thirty-five fluorescent images along the *z* axis with 80 nm intervals (optical sections) were obtained using Zeiss Axio Imager Z2 upright fluorescent microscope via a 63×/1.40 NA objective and deconvolved using AxioVision 4.9 software. PLA reaction products appear as green foci, which originate from the location of ErbB2 interaction with its partners. The number of green foci was counted using Adobe Photoshop.

### RNA Fluorescence In Situ Hybridization

Custom Stellaris RNA fluorescence in situ hybridization (FISH) Probes were designed against mouse Nrg1 (NM_178591.2) by using the Stellaris RNA FISH Probe Designer (Biosearch Technologies, Inc, Petaluma, CA) available online at www.biosearchtech.com/stellarisdesigner. The gastrocnemius muscle sections were hybridized with the mouse Nrg1 Stellaris FISH Probe set labeled with CAL Fluor Red 590 dye following the manufacturer’s instructions. Alexa Fluor 488-conjugated isolectin GS-IB4 was incorporated in hybridization mix to label capillaries. Fluorescent images along the *z* axis with 80 nm intervals (optical sections) were obtained using Zeiss Axio Imager Z2 upright fluorescent microscope via a 63×/1.40 NA objective and deconvolved using AxioVision 4.9 software.

### EPR Spectrometry (Quantitation of NO)

NO formation by isolated left coronary artery (LCA) was detected by EPR spectroscopy using NO spin trap Fe^2+^-(N-methyl-D-glucamine dithiocarbamate)_2_ (Fe-MGD).^33,39^ After appropriate treatments, LCA was dissected out from mouse heart, longitudinally opened and washed with MEM. LCA was incubated in 50 µL of MEM containing 10 µmol/L ACh, 0.1 mmol/L sodium ascorbate, and 2 mmol/L Fe-MGD. NO generated by LCA was detected as paramagnetic NO-Fe^2+^-MGD_2_ adduct using Bruker EMX nano spectrometer at room temperature. EPR spectra were acquired under following scan conditions: microwave frequency, 9.88 GHz; power, 50.24 mW; attenuation 6 dB; modulation frequency, 100 kHz; modulation amplitude, 3.906 G; sweep time, 30 s; time constant, 0.1 s; receiver gain, 20 dB; and magnetic field, 3400 to 3500 G.

### Superoxide Anion Detection and Quantitation

Superoxide formation in HCAEC or gastrocnemius tissue from mice was detected by EPR spectroscopy using spin probe CMH.^44^ After appropriate treatments, HCAECs were resuspended in Krebs-Henseleit Buffer (118 mmol/L NaCl, 4.7 mmol/L KCl,1.2 mmol/L MgSO_4_, 1.25 mmol/L CaCl_2_, 1.2 mmol/L KH_2_PO_4_, 25 mmol/L NaHCO_3_, 11 mmol/L glucose, pH 7.4) and incubated with 1 mmol/L CMH in presence of 25 μmol/L desferoxamine and 2.5 μmol/L diethyldithiocarbamate at 37 °C. In case of mouse tissue, after subjecting mice to hindlimb RIPC treatments, gastrocnemius tissue was dissected out from hindlimb, cut into 1 mm cubes and 25 mg tissue was incubated in 100 µL Krebs-Henseleit buffer containing 1 mmol/L CMH, 25 μmol/L desferoxamine and 2.5 μmol/L diethyldithiocarbamate. Superoxide generated by HCAECs or mouse tissue was detected as paramagnetic nitroxide radical (CM*) using Bruker EMX Micro spectrometer at room temperature. EPR spectra were acquired under following scan conditions: microwave frequency, 9.835 GHz; power, 20 mW; attenuation 10 dB; modulation frequency, 100 kHz; modulation amplitude, 1.00 G; sweep time, 60 s; time constant, 10.24 s; receiver gain, 30 dB; magnetic field, 3470 to 3540 G.

### eNOS Activity Assay

eNOS activity was assayed using Cayman’s NOS activity assay kit (catalog No. 781001) following manufacturer’s instruction. Briefly, after appropriate treatments, HCAECs were washed with PBS containing 1 mmol/L EDTA and lysed in homogenization buffer (25 mmol/L Tris-HCl pH 7.4, 1 mmol/L EDTA, and 10 mmol/L EGTA) by brief sonication. eNOS activity was assayed using 20 μg protein from the cell lysate in 50 μL reaction buffer (25 mmol/L Tris-HCl pH 7.4, 3 μmol/L tetrahydrobiopterin, 1 μmol/L flavin adenine dinucleotide, and 1 μmol/L flavin adenine mononucleotide) containing 1 mmol/L NADPH, 1 μCi [^3^H]-arginine, 0.6 mmol/L CaCl_2_ and 0.1 μmol/L calmodulin at 37 °C for 20 minutes. In some cases, 1 μmol/L L-NNA was used to inhibit eNOS activity. At the end of incubation period, the assay was stopped by adding 400 μmol/L stop buffer (50 mmol/L HEPES pH 5.5, 2 mmol/L EDTA). Unused arginine was removed by the resin supplied by the manufacturer, and L-citrulline formed during the assay eNOS was quantitated from the eluate using a liquid scintillation counter and the specific activity was calculated.

### Statistics

All the cell culture-based experiments were repeated 3×, and data are presented as mean±SEM. The statistical significance of the results was analyzed by Student *t* test or 1-way ANOVA followed by Tukey post test was performed using GraphPad -Prism software (version 8), and *P* values±0.05 were considered statistically significant. Animal experiments were conducted with a group of 4 to 5 mice per treatment. For all cell culture studies, we have repeated the experiments with a minimum of three replicates (n=3), unless mentioned otherwise. In the case of Western blot analysis, RT-PCR, immunofluorescence staining, and NO spin trapping, one set of data is presented. The normality and variance were not tested to determine whether the applied parametric test were appropriate.

## Results

### Superoxide Anion Dependent Nrg1β Release in RIPC Protects Against MI

**Figure 1.**
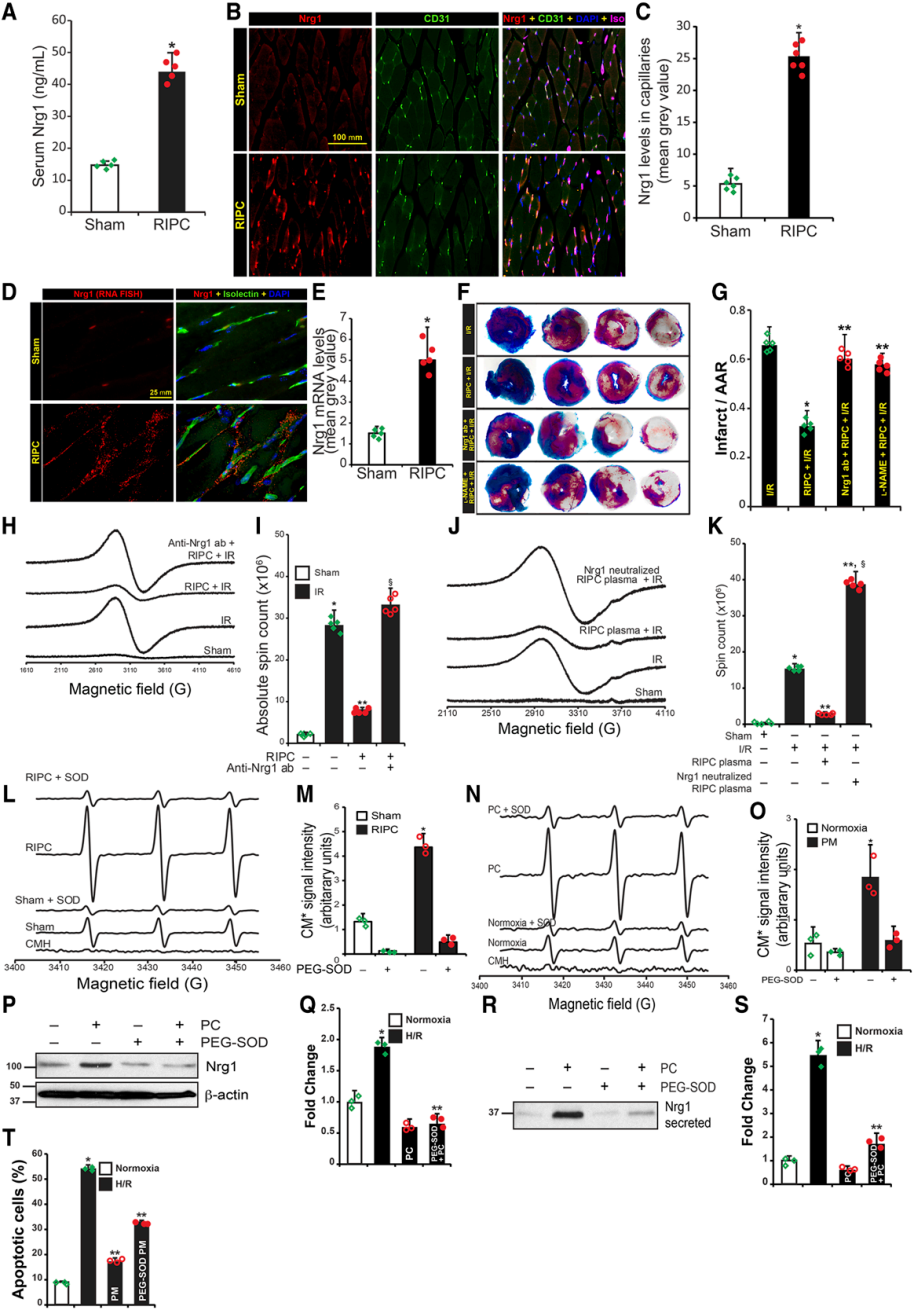
**Superoxide-induced Nrg1 (neuregulin 1) release by the microvascular endothelial bed of the hindlimb acts as remote ischemic preconditioning (RIPC)-factor and protects the myocardium from ischemia/reperfusion (I/R)-induced injury. A**, Mice were either subjected to sham or three cycles of 5 min hindlimb ischemia followed by 5 min reperfusion. Thirty minutes after the last I/R cycle, serum Nrg1 levels were determined by ELISA. Mean±SEM was plotted as a bar graph (n=5). *P<0.01 vs sham. **B**, Formaldehyde fixed paraffine embedded gastrocnemius muscle sections from sham or RIPC-treated mice were immunostained with anti-Nrg1 and anti-CD31 antibodies along with isolectin GS-IB4, scale bar=100 mm. **C**, Nrg1 specific fluorescent signals were quantitated, and mean±SEM fluorescent values were plotted as a bar graph (n=6). *P<0.01 vs sham (Student *t* test). **D**, To confirm the source of Nrg1, gastrocnemius muscle sections were hybridized with mouse Nrg1 Stellaris fluorescent in situ hybridization (FISH) Probe set labeled with CAL Fluor Red 590 Dye. Capillaries in the sections were stained with isolectin GS-IB4 conjugated with Alexa Fluor 488. Scale bar=25 mm. **E**, Mean fluorescence values from RNA FISH was quantitated, and mean±SD plotted as a bar graph (n=5). *P<0.01 vs sham. **F**, Mice were treated with control IgG or with neutralizing Nrg1 antibodies or l-NAME subjected to either I/R surgery or RIPC followed by I/R surgery, and TTC staining was performed. **G**, Infarct area in relation to area at risk (AAR) was calculated, and mean±SD plotted as a bar graph (n=5). *P<0.01 vs I/R, **P<0.01 vs RIPC+I/R (ANOVA). **H**, Mice were subjected to sham or myocardial I/R or RIPC followed by I/R surgery and perfused with annexin-V-Fe complex as described in Methods. The infarcted tissue was excised, and tissue bound annexin-V was quantified by measuring annexin-V conjugated paramagnetic iron by electron spin resonance (EPR). To block the function of circulating Nrg1 in mice, neutralizing anti-Nrg1 antibodies (150 µg/kg) or control IgG was administered to mice before RIPC and then subjected to RIPC+IR injury. **I**, Absolute spin count was calculated from EPR spectra, and means±SD (n=5) was plotted as a bar graph. *P<0.01 vs sham, **P<0.01 vs IR, §*P*<0.01 vs RIPC+IR, (ANOVA). **J**, Plasma from RIPC-subjected mice were prepared. Sibling mice were administered with RIPC, or Nrg-1 neutralized RIPC-plasma and then subjected to IR. Tissue apoptosis was measured as in **H. K**, Tissue bound annexin-V was quantified, and mean±SD was plotted as a bar graph (n=5). *P<0.01 vs sham, **P<0.01 vs IR, §*P*<0.01 vs RIPC+IR (ANOVA). **L**, Superoxide formation was quantitated in gastrocnemius tissue from sham or RIPC-exposed mice. Superoxide formation isolated was determined by EPR using 1-hydroxy-3-methoxycarbonyl-2,2,5,5-tetramethylpyrrolidine hydrochloride (CMH). **M**, CM* EPR signals were quantitated by Spin Count using Xenon nano 1.2 software and plotted as a bar graph (n=3). *P<0.01 vs sham (ANOVA). **N**, HMVECs were exposed to hypoxia for 2 h, followed by reoxygenation for 1 h (PC, preconditioning), and superoxide formation in HCAECs was determined by EPR using CMH. **O**, CM* EPR signals were quantitated and plotted as a bar graph (n=3). *P<0.01 vs normoxia (ANOVA). **L–O**, PEG-SOD (polyethylene glycol-superoxide dismutase) was added to determine the superoxide-specific EPR signal. **P**, HMVECs (n=3) were incubated with or without PEG-SOD for 6 h and subjected to PC. Cell lysates were analyzed for full-length Nrg1; (**Q**) Densitometry of Figure 1P, *P<0.05 vs untreated, **P<0.01 vs PC. **R**, Detection of Nrg1in culture medium from control or PC-subjected cells. Medium was analyzed for the full length and secreted Nrg1, respectively, by Western blotting, *P<0.01 vs HR, **P<0.01 vs PEG-SOD. **T**, HCAECs were incubated with preconditioned medium (PM) obtained from control or PEG-SOD treated HMVECs. Then they were exposed to hypoxia/reoxygenation (H/R). Cells undergoing apoptosis were labeled with annexin V-FITC conjugate, and the percentage of apoptotic cells was quantitated by fluorescence-activated cell sorting analysis using Attune NxT Flow Cytometer and plotted as a bar graph (n=3). *P<0.01 vs normoxia, **P<0.01 vs PM+H/R (ANOVA, Tukey post test).

Since Nrgs are secreted by endothelial cells in response to I/R of the heart,^13^ we determined whether RIPC would induce Nrg1β expression in a remote vascular bed that is subjected to short-episodes of I/R. Hindlimb in mouse has widely been used as a remote vascular bed.^37,38,45^ Additionally, ischemic cycles of lasting 2 minutes was found to offer same protection as cycles of 5 minutes ischemia followed by 5 minutes reperfusion. However, prolonged cycles lasting 10 minutes abrogated protection.^38^ Further, RIPC of either one hindlimb or both limbs in mice were equally protective.^38^ Based on the previous established cycles for mice, we used 5 minutes ischemia followed by 5 minutes reperfusion in our experiments.^37,38^ We found that circulating Nrg1β level was increased by 3-fold due to IPC of the FA (Figure 1A). Next, to identify the source of circulating Nrg1β, we probed gastrocnemius muscle sections from RIPC subjected mice for Nrg1 by immunofluorescence. Nrg1β level was increased by 5-fold in RIPC-treated mice (Figure 1B and 1C). Nrg1β was specifically localized in the microvascular endothelial cells, as confirmed by its co-localization with CD31 and isolectin positive cells (Figure 1B and Figure I in the Data Supplement). Since Nrg1β is a known paracrine secreted factor,^46^ we confirmed its expression in microvascular endothelial cells of gastrocnemius tissue using RNA FISH approach. As shown in Figure 1D, FISH signals were localized in and around microvasculature, confirming microvascular endothelial cells as the source of RIPC-induced Nrg1β. Compared with the sham-treated group, the RIPC-group showed a 3-fold higher level of Nrg1β mRNA (Figure 1D and 1E). Further, we determined whether microvascular endothelial cells would secrete Nrg1β during short I/R episodes similar to that applied in RIPC. We exposed HMVECs to different periods of hypoxia, followed by an hour of reoxygenation at normoxic conditions (referred to as preconditioning or preconditioning hereafter) and determined changes in Nrg1β mRNA levels by RT-PCR. Preconditioning ranging from 30 minutes to 2 hours induced the expression of Nrg1β by 2- to 4-folds in HMVECs (Figure IIA in the Data Supplement). Similarly, immunoblotting of proteins extracted from control and preconditioned HMVECs showed a 3- to 6-fold induction of full-length Nrg1 (Figure IIB in the Data Supplement). However, Nrg1β levels began to decrease after 2 hours onwards. We further determined whether the release of Nrg1β to the extracellular medium by HMVECs could account for the decrease in Nrg1β protein levels. Immunoblot analysis of culture medium collected from precondition exposed HMVECs demonstrated increased levels of Nrg1β in the culture media in response to preconditioning (Figure IIC in the Data Supplement). Since Nrg1β is well documented to protect against MI in animal models,^19^ we determined whether the RIPC-mediated release of Nrg1β would decrease MI. As shown in Figure 1F, I/R caused a significant increase in AAR and MI, as determined by TTC-staining in sham-treated mice; in contrast, there was a significant reduction in both AAR and MI in mice that underwent RIPC before I/R (Figure 1G). To confirm whether Nrg1β is the major factor in RIPC that afforded protection, we injected neutralizing-Nrg1β antibody to mice before RIPC and I/R. As shown in Figure 1F and 1G, the protective effect of RIPC was significantly decreased in mice injected with Nrg1 neutralizing antibody compared with mice treated with control IgG. Since earlier studies in our laboratory^33^ demonstrated an essential role of eNOS-mediated NO release in the protection against I/R-induced MI, we tested whether eNOS plays any role in RIPC-mediated protection of MI. Surprisingly, inhibition of eNOS activity by L-NAME abolished RIPC-afforded protection of heart from MI (Figure 1F and 1G). TTC staining does not directly quantify whole tissue apoptosis in the heart. Therefore, we determined the myocardial apoptosis of entire infarcted area using EPR spectrometry with magnetic annexin-V beads, as described in the methods. As shown in Figure 1H, I/R significantly increased apoptosis of the entire infarcted area, but RIPC significantly decreased the level of apoptosis (Figure 1I). The Nrg1-neutralizing antibody abolished the protective effect of RIPC in contrast to IgG controls, as demonstrated by decreased apoptosis (Figure 1H and 1I). The generation of humoral factors by RIPC is confirmed by the transferability of RIPC-benefits via serum or plasma from RIPC-treated subjects to naïve subjects.^8,47^ Hence, to confirm Nrg1β released into the circulation as the RIPC factor, we treated plasma from RIPC-treated animals with neutralizing Nrg1 antibody and then transferred to naïve mouse before I/R injury. While RIPC-plasma transfer conferred significant protection in naïve mice against I/R-induced tissue apoptosis (Figure 1J and 1K), Nrg1-neutralized plasma from RIPC-mice not only abolished the protection, but also resulted in two-fold higher tissue apoptosis (Figure 1K).

Since I/R induces the generation of ROS,^48^ we hypothesized that RIPC would produce O_2_^•−^ that may induce Nrg1β expression in RIPC. As shown in Figure 1L and 1M, RIPC induced the production of O_2_^•−^ in the gastrocnemius tissue of mice in I/R. Likewise, HMVECs produced a significant amount of O_2_^•−^ in response to preconditioning (Figure 1N and 1O). In both cases, PEG-SOD1 (polyethylene glycol conjugated superoxide dismutase) abrogated CMH spectra (Figure 1L and 1N). Since CMH does not discriminate O_2_^•−^ and hydroxyl radicals (^·^OH), we used PEG-SOD to confirm the species of ROS generated by RIPC. PEG-SOD1 decreased the CMH spectra, indicating O_2_^•−^ is produced in RIPC. In addition, removal of O_2_^•−^ by PEG-SOD1 reduced the level of both, full-length Nrg1β and secreted Nrg1β by HMVECs (Figure 1P through 1S, respectively). To identify the mechanism by which RIPC-mediated O_2_^•−^ induces Nrg1β expression, we used pharmacological inhibitors to block activation of MAPK and analyzed preconditioning-induced Nrg1β expression in HMVECs. Inhibition of ERK resulted in the blockade of preconditioning-induced Nrg1β expression (Figure IID in the Data Supplement). If RIPC-mediated Nrg1β was induced by O_2_^•−^ then removal of O_2_^•−^ by SOD should abolish the protective effect of RIPC. As shown in Figure 1T and Figure IIE in the Data Supplement, pretreatment of HCAECs with PM obtained from PEG-SOD treated HMVECs did not protect HCAECs from H/R-induced apoptosis compared with the exposure of HCAECs cells with untreated PM. Collectively, these studies demonstrate that RIPC induces the production of O_2_^•−^ that mediate Nrg1β production and secretion, which is a critical RIPC agent required for protection against MI in mice.

### RIPC Protected Myocardial Perfusion via Nrg1-Dependent eNOS Activation

**Figure 2.**
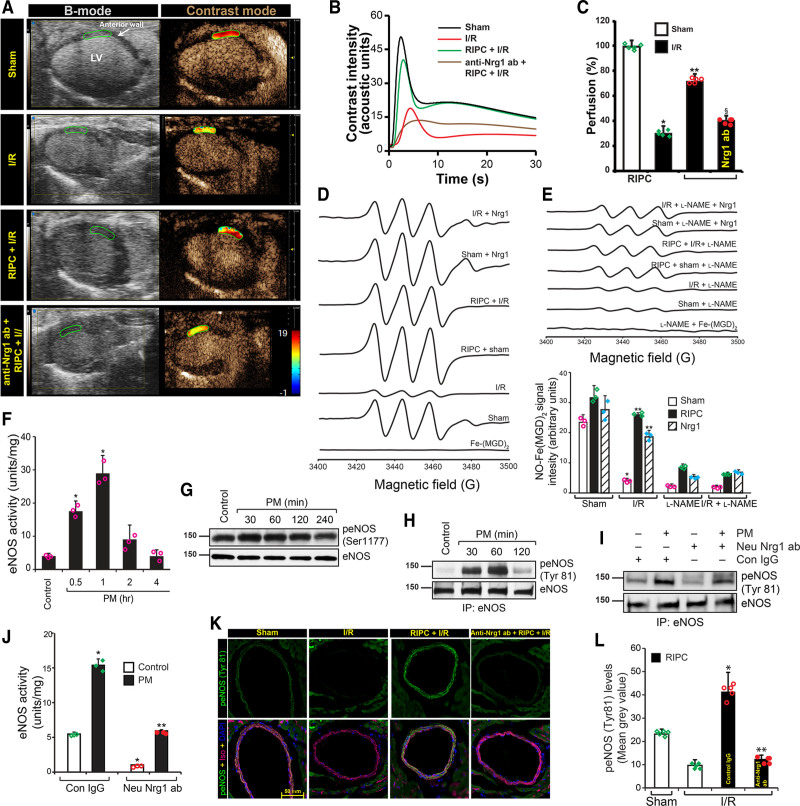
**Remote ischemic preconditioning (RIPC) improved cardiac perfusion via Nrg1 (neuregulin 1)-dependent eNOS (endothelial nitric oxide synthase) activation. A**, Mice were subjected to sham or myocardial ischemia/reperfusion (I/R) or RIPC+I/R surgery, and at the end of the reperfusion period, the nontargeted contrast agent was injected to mice via femoral vein while collecting B-mode/contrast mode images of the heart from long-axis view using Vevo 3100. To block the function of circulating Nrg1 in mice, neutralizing anti-Nrg1 antibodies (150 µg/kg) was administered to mice before RIPC and then subjected to RIPC+I/R injury. **B**, Contrast intensities in the left ventricle (LV) anterior wall downstream to the ligation site were quantitated and plotted over time. Loss of peak height and the rightward shift of peak indicates hypoperfusion. **C**, From the rate of contrast agent intensity change, the perfusion index was calculated. Percentage of perfusion was calculated based on the perfusion index assuming its level in the sham group is 100% and blotted as a bar graph (n=5) and shown as a heat map over the anterior LV in **A**. Values are means±SEM (n=5 mice). *P<0.01 vs sham, **P<0.01 vs I/R, §*P*<0.01 vs RIPC+I/R (ANOVA). **D**, Left coronary artery from mice were isolated, and after indicated treatments, NO formation was detected by electron spin resonance (EPR) spectrometry using NO spin trap Fe2+-(N-methyl-D-glucamine dithiocarbamate) 2 (Fe-[MGD]2) as described in methods. **E**, NO-Fe(MGD) 2 spin count was quantified and plotted as a bar graph (n=5). *P<0.01 vs sham; **P<0.01 vs I/R (ANOVA). **F–H**, HMVECs were exposed to 2 h hypoxia followed by 1-hour reoxygenation, and the medium was collected (preconditioned medium [PM]). HCAECs were treated with PM for the indicated period, lysed, and cell lysates were collected. An equal amount of cell lysates were analyzed for eNOS activity (**F**) and plotted as a bar graph (n=3). *P<0.01 vs control (ANOVA). An equal amount of cell lysates were analyzed for eNOS phosphorylation at Ser 1177 by Western blotting using its phospho-specific antibodies and normalized with total eNOS levels (**G**), or the cell lysates were immunoprecipitated with anti-eNOS antibodies, and the immunoprecipitates were analyzed by Western blotting using anti-phospho-tyrosine specific antibodies (**H**). **I–J**, To determine the role of HMVEC-released Nrg1 in PM in the activation of eNOS, the collected PM was pretreated with neutralizing anti-Nrg1 antibodies and incubated with human coronary artery endothelial cell (HCAEC) for 1 h, and cell lysates were collected and analyzed for eNOS tyrosine phosphorylation by Western blotting (**I**) or eNOS activity (**J**). **K**, Mice were injected control IgG or neutralizing anti-Nrg1 antibodies via the tail vein. After 24 h, they were subjected to hindlimb RIPC followed by myocardial I/R, and heart from these mice was sectioned below the LAD ligation point and analyzed for eNOS phosphorylation at Tyr81 by immunofluorescence staining. Scale bar=50 mm. **L**, Green fluorescence localized to the coronary artery endothelial cells were quantitated (n=5), and the results were plotted as a bar graph. *P<0.05 vs I/R, **P<0.05 vs control IgG+I/R (ANOVA).

Prolonged hypoperfusion of myocardium has a direct impact on cardiac tissue apoptosis^49^ and I/R-insult results in subsequent impairment in myocardial perfusion leading to the development of MI during recovery.^50^ Because neutralization of Nrg1β blocked RIPC-induced attenuation of MI, we determined myocardial perfusion of mice subjected to I/R using contrast echocardiography to demonstrate whether RIPC would improve myocardial perfusion. As shown in Figure 2A, myocardial perfusion was significantly impaired (≈60%) in I/R treated mice but RIPC significantly protected against such impairment. In contrast, Nrg1β neutralizing antibody abolished the protective effect of RIPC-mediated myocardial perfusion, demonstrating Nrg1β -dependent protection is critical in improved myocardial perfusion due to RIPC. The difference in contrast intensity over time shows rate of perfusion of the myocardium. Loss of peak height in I/R-subjected mice indicates reduced perfusion and rightward shift of the peak shows delay in perfusion, which in turn, point to vascular dysfunction (Figure 2B). RIPC restored perfusion and attenuated vascular dysfunction (Figure 2B and 2C). Taken together, these data demonstrate a pivotal role of Nrg1β in improved myocardial perfusion and consequent protection of vascular function from I/R injury.

It is well established that vascular endothelium is the main source of vasoactive factors that regulate vascular tone in mammals.^51^ Since eNOS-derived NO plays a central role in the regulation of vascular tone of coronary artery,^52^ we speculated an impairment in NO generation by coronary artery in I/R. We measured NO generation using EPR and as shown in Figure 2D and 2E, LCA from mice subjected to I/R demonstrated about 80% loss of NO production in response to ACh. However, this decrease in NO was restored to a considerable extent in mice that underwent RIPC (Figure 2E). Further, LCA preincubated with recombinant Nrg1β and then exposed to I/R ex vivo showed 70% to 80% restoration in NO generation (Figure 2D and 2E). In addition, both Nrg1-sensitive RIPC, as well as direct Nrg1 mediated enhancement of NO generation by LCA was blocked by its inhibitor l-NAME (Figure 2D and 2E). If Nrg1β was the active agent in RIPC that rescued NO generation in LCA of I/R subjected mice then treatment of mice with neutralizing Nrg1β antibody before RIPC should abrogate the protective effect of RIPC mediated by NO generation. Indeed, as shown in Figure 2D and 2E, treatment of mice with neutralizing-Nrg1β antibody caused a significant decrease in the NO generation in the coronary endothelium. Taken together, these results establish that Nrg1β is a critical factor in RIPC that restores NO generation in I/R-impaired endothelium.

Since NO generated by eNOS is critically important for the protection against endothelial dysfunction of coronary artery in I/R, we determined the mechanism by which Nrg1 might induce NO production. We first recapitulated the in vivo conditions in I/R using a cell culture model. We incubated HCAECs with PM and analyzed eNOS activity. A significant increase (4–6-fold) in the activity of eNOS was observed in HCAEC treated with PM (Figure 2F). Since eNOS activation could be regulated by 2 major activating phosphorylations, Ser1177 and Tyr81,^30,53^ we determined phosphorylation state of eNOS in HCAEC. As shown in Figure 2G, eNOS phosphorylation at Ser1177 was marginally increased in response to PM, in contrast to activation of eNOS as shown in Figure 2F. Surprisingly, PM treatment caused 10-fold increase in tyrosine phosphorylation of eNOS (Figure 2H), suggesting a major role of Tyr81 in the activation of eNOS. Next, we evaluated whether Nrg1 is the vasoactive component of PM that is secreted by HMVEC during preconditioning that activates eNOS. We incubated HCAEC with PM containing neutralizing anti-Nrg1β antibody and determined eNOS tyrosine phosphorylation and eNOS enzyme activity. As shown in Figure 2I and 2J, neutralizing-Nrg1 antibody in PM attenuated eNOS tyrosine phosphorylation and eNOS activity, in contrast to control IgG demonstrating that Nrg1β is the major component in PM that induces eNOS activation. Next, to verify eNOS activation by RIPC in myocardium, we probed the heart sections from control and RIPC-treated mice subjected to I/R with antiphospho-eNOS (Tyr81) antibody. As shown in Figure 2K and 2L, eNOS phosphorylation on Tyr81 was induced by RIPC and it was localized only around major artery endothelial cells, such as LCA branches. Pretreatment of mice with neutralizing Nrg1 antibody reduced eNOS-Tyr81 phosphorylation to basal levels in contrast to control IgG (Figure 2K and 2L).

### Endothelial ErbB2 Is Required for RIPC/Nrg1β-Mediated Cardiac Protection

**Figure 3.**
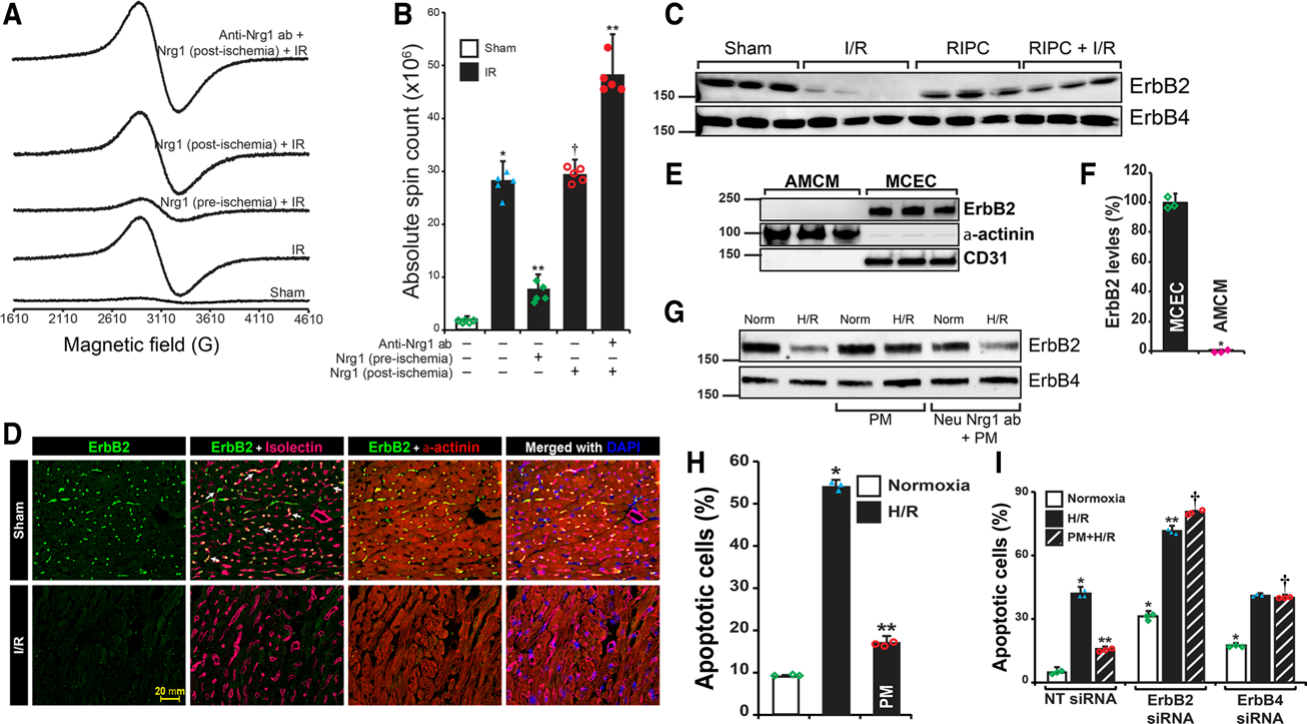
**Only preischemic and not postischemic Nrg1β (neuregulin 1) administration protects myocardium due to loss of endothelial-ErbB2 (Erb-B2 receptor tyrosine kinase 2) during ischemia. A**, To determine the protective function of Nrg1 during ischemia or reperfusion, recombinant Nrg1β (4 µg/kg) was injected into mice before ischemia (preischemia) or after ischemia (postischemia) but before reperfusion. After ischemia/reperfusion (I/R), the heart was isolated and perfused with annexin-V-Fe complex. The infarcted tissue was excised, and tissue bound annexin-V was quantified by electron spin resonance (EPR). **B**, The absolute spin count was calculated from the EPR spectra of paramagnetic-Fe bound to annexin-V and plotted as a bar graph. Values are means±SD (n=5 mice). *P<0.01 vs sham; **P<0.01 vs I/R, †*P*<0.01 vs Nrg1 (preischemic) plus IR (ANOVA). **C**, Mice were subjected to sham, I/R, remote ischemic preconditioning (RIPC) or RIPC+I/R, and the infarcted tissue region was excised. Protein extract was prepared from the excised tissue and analyzed by Western blotting for ErbB2 and ErbB4 levels. **D**, Sections of sham and I/R mouse heart were subjected to immunofluorescence staining using anti-ErbB2 and anti-α-actinin (cardiomyocyte marker) antibodies. Isolectin-Alexa Fluor 647 conjugate was used to stain endothelial cells selectively. Fluorescent images of the stained sections were obtained using an upright Zeiss fluorescence microscope (AxioImager Z2) via 40×/1.4 NA objective. Scale bar=20 mm. **E** and **F**, Adult mouse cardiomyocytes and mouse cardiac endothelial cells were isolated from adult mouse heart, and cell lysate was prepared. An equal amount of protein from cell lysates was analyzed for ErbB2 levels by Western blotting. Blot was reprobed for α-actinin and CD31 (**F**). ErbB2 levels were quantified and plotted as a bar graph (n=3). *P<0.01 vs MCEC (Student *t* test). **G**, HCAECs were pretreated with preconditioned medium (PM) or Nrg1-neutralized PM, then exposed to hypoxia/reoxygenation (H/R), and cell lysates were prepared. An equal amount of protein from each sample was analyzed for ErbB2 and ErbB4 levels by Western blotting. **H**, HCAECs were pretreated with or without PM and exposed to H/R. At the end of treatment, cells undergoing apoptosis were labeled with annexin V-FITC conjugate, and the percentage of apoptotic cells was quantitated by fluorescence-activated cell sorting analysis using Attune NxT Flow Cytometer and plotted as a bar graph (n=3). *P<0.01 vs normoxia, **P<0.01 vs H/R (ANOVA). **I**, HCAECs were transfected with nontarget (NT) or ErbB4 or ErbB4 siRNA (100 nmol/L), and after recovery from transfection, they were treated with or without PM and exposed to H/R. Cells undergoing apoptosis were labeled with annexin V-FITC conjugate, and the percentage of apoptotic cells was quantitated and plotted as a bar graph (n=3). *P<0.01 vs NT siRNA normoxia, **P<0.01 vs NT siRNA H/R; †*P*<0.01 vs NT siRNA PM+H/R (ANOVA). ANOVA followed by Tukey post test was performed using GraphPad-Prism software (version 8).

Since Nrg1-neutralizing antibody abrogated the beneficial effect RIPC in MI, we evaluated whether direct administration of Nrg1β would protect the myocardium from I/R injury. We injected rhNrg1β via tail vein before ischemia and after ischemia, but before the reperfusion and determined the level of myocardial apoptosis using EPR-based assay. As demonstrated in Figure 3A and 3B, administration of Nrg1β to mice preischemia reduced I/R-induced myocardial apoptosis similar to protection afforded by RIPC. Importantly, postischemia injection of rhNrg1β did not yield any protection against MI in I/R (Figure 3A and 3B), demonstrating loss of a critical factor during ischemia that is essential for the protective effect of Nrg1β or RIPC against I/R-induced MI. Additionally, injection of Nrg1-neutralizing antibody postischemia resulted in increased tissue apoptosis (Figure 3A and 3B). Neuregulins bind to ErbB tyrosine kinase receptors expressed on cardiomyocytes and activate cell survival signaling in these cells.^54^ Nrg1 signaling in the adult heart is mediated by ErbB2/ErbB4 heterodimers,^23^ which modulates cardiomyocyte contractile functions.^22^ Based on this information, we determined the level of ErbB2 or ErbB4 in the heart during I/R injury. Interestingly, we found an acute decrease of ErbB2 expression in I/R in the myocardium (Figure 3C), but the ErbB4 level remained unaffected. In addition, we found that mice subjected to RIPC had normal levels of ErbB2 (Figure 3C). These studies demonstrate that protection of ErbB2 against degradation by RIPC or Nrg1 during I/R is a major mechanism in RIPC-mediated decrease in MI. It is important to note here that cardiomyocytes make up 70% to 85% of heart by volume but endothelial cells constitute up to 64% of total cell population in the mammalian heart.^55^ We determined whether there is a cell-specific loss of ErbB2 in I/R. Sections of heart tissue from sham and I/R-subjected mice were immunostained for ErbB2 along with endothelial and cardiomyocyte markers. Immunofluorescence images from the sections showed significant decrease in the expression of ErbB2 only in endothelial cells, but not in cardiomyocytes, which were marked by α-actinin (Figure 3D). These studies show that I/R resulted in the loss of endothelial ErbB2 (Figure 3D). Although the interaction of endothelial-secreted Nrg1β with cardiomyocyte ErbB2 is well established,^17–19^ the interaction of adult murine cardiomyocyte ErbB2 with Nrg1β is poorly understood. Studies have shown that murine ErbB2 expression is decreased by almost 99% in the cardiomyocytes at postnatal day 7, and its further decline at P28.^20^ Additionally, severe loss (95%) of ErbB2 in the cardiomyocytes of mice at 12 weeks of age has been demonstrated.^21^ We found that adult murine cardiomyocytes do not express ErbB2 as presented in Figure 3E and 3F. In contrast, isolated endothelial cells from adult mouse heart do express high levels of ErbB2, suggesting a critical role of endothelial ErbB2 in I/R. In addition, we found that ErbB2 was degraded in HCAEC subjected to H/R (Figure 3G). However, treatment of HCAEC with PM prevented ErbB2 degradation (Figure 3G). In contrast, treatment with neutralizing-Nrg1 antibody abolished the protection afforded by PM. As shown in Figure 3H and Figure IIIA in the Data Supplement, H/R caused apoptosis in >50% of HCAECs, but pretreatment with PM protected HCAECs from H/R-induced apoptosis (Figure 3H). If ErbB2 was critically required for Nrg1 or RIPC-mediated protection of apoptosis, then depletion of ErbB2 in the endothelial cells should result in significant apoptosis in H/R. As shown in Figure 3I and Figure IIIB in the Data Supplement depletion of ErbB2 resulted in loss of protection with increased endothelial apoptosis in H/R. Taken together, we show that Nrg1β mediated inhibition of endothelial ErbB2 degradation in I/R is a major mechanism of RIPC-mediated protection.

### RIPC-Mediated Protection of Endothelial-ErbB2 Is Essential to Prevent the Loss of Trx2 and Endothelial Cell Apoptosis

**Figure 4.**
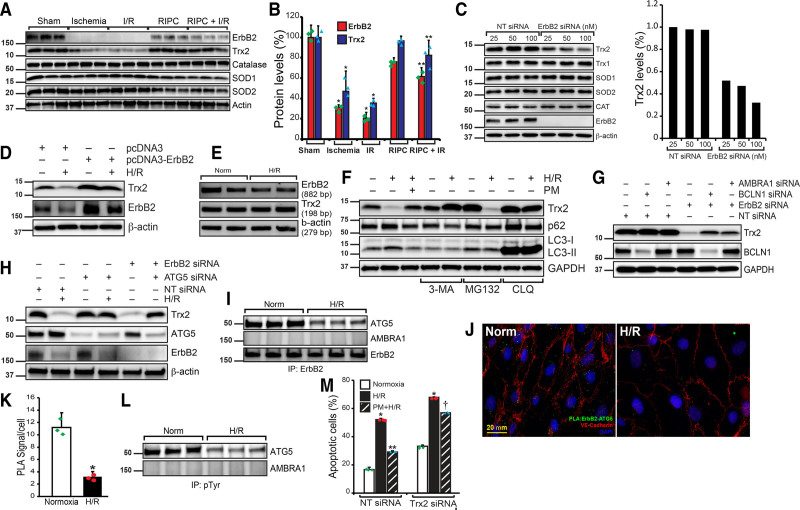
**Remote ischemic preconditioning (RIPC) protects ErbB2 (Erb-B2 receptor tyrosine kinase 2) from ischemia/reperfusion (I/R)-mediated degradation and the loss of ErbB2 results in Trx2 (thioredoxin 2) autophagy and apoptosis. A**, Mice were subjected to a sham procedure, 30 min ischemia, I/R, RIPC or RIPC+I/R, and the infarcted tissue region were excised. Protein extract was prepared from the excised tissue and analyzed by Western blotting for ErbB2, Trx2, catalase, SOD1, SOD2, and total actin. **B**, ErbB2 and Trx2 levels in the blots were quantified and plotted as a bar graph (n=3). *P<0.01 vs sham; **P<0.01 vs I/R. **C**, HCAECs were transfected with nontarget (NT) or ErbB2 siRNA (25, 50, 100 nmol/L). After 48 h of transfection, cell lysates were prepared, and an equal amount of protein from each cell lysate was analyzed for Trx1, Trx2, SOD1, SOD2, and catalase levels using their specific antibodies. Blots were reprobed with anti-ErbB2 and anti-β-actin antibodies to determine the depletion of ErbB2 and equal protein loading, respectively. Bar graph shows the level of Trx2. **D**, HCAECs were transfected with pcDNA3 empty or pcDNA3-ErbB2 plasmids. After 24 h of transfection, cells were exposed to hypoxia/reoxygenation (H/R), and cell lysates were prepared. An equal amount of protein from each sample was analyzed for Trx2 and ErbB2 levels using their specific antibodies. Blots were reprobed with anti-β-actin antibodies. **E**, HCAECs were exposed to normoxia or H/R, mRNA was isolated, and cDNA was synthesized. PCR was performed to determine levels of ErbB2 (primers, 5′- GTGCTGGTCAAGAGTCCCAACCATG-3′ and 5′- ATCTGGCTGGTTCACATATTCAGGC-3’), Trx2 (primers, 5′- CCACACCTTGGTCCTCATCT-3′ and 5′- AGGAGGTGGAAGGGATGACT-3′), and β-actin (primers, 5′-CCGCCAGCTCACCAT-3′ and 5′-GTGTGGTGCCAGATTTTCTC-3′). **F**, HCAECs were treated with 3-methyl adenine (3-MA), MG132 (proteasomal inhibitor), or chloroquine (CLQ) before exposing them to H/R. After the treatment, cells were lysed, and the cell lysates were analyzed by Western blotting for Trx2, p62, microtubule-associated protein 1, light chain 3-I/II level using its specific antibody, and the blot was reprobed for β-actin for normalization. **G**, HCAECs were transfected with Ambra1 or beclin 1 (BCLN1) siRNA in combination with NT or ErbB2 siRNA (100 nmol/L). After 48 h of transfection, cell lysates were prepared, and an equal amount of protein from each cell lysate was analyzed for Trx2, beclin 1, and GAPDH levels using their specific antibodies. **H**, HCAECs were transfected with NT or ATG5 (autophagy-related 5) siRNA and exposed to H/R or transfected with ATG5 siRNA and ErbB2 siRNA. After 48 h of transfection and H/R treatment, cell lysates were prepared, and an equal amount of protein from each cell lysate was analyzed for Trx2, ATG5, ErbB2, and GAPDH levels using their specific antibodies. **I**, HCAECs were exposed to normoxia or H/R, and cell lysates were immunoprecipitated with anti-ErbB2 antibodies. Immunoprecipitates were analyzed for ATG5, AMBRA1 (activating molecule in beclin 1-regulated autophagy), and ErbB2. **J**, To study the in situ interaction of ErbB2 and ATG5, HCAECs were subjected to H/R and proximity ligation assay (PLA) was performed using anti-ErbB2 and anti-ATG5 antibodies. Scale bar=20 mm. **K**, Green foci-proximity signals of ErbB2 and ATG5 association were counted and plotted as a bar graph. *P<0.01 vs normoxia. Student *t* test. **L**, HCAECs were exposed to normoxia or H/R, and cell lysates were immunoprecipitated with anti-pTyr antibodies. Immunoprecipitates were analyzed for ATG5 and AMBRA1. **M**, HCAECs were transfected with NT or Trx2 siRNA (100 nmol/L), and after recovery from transfection, they were treated with or without preconditioned medium (PM) and exposed to H/R. Cells undergoing apoptosis were labeled with annexin V-FITC conjugate, and the percentage of apoptotic cells was quantitated and plotted as a bar graph (n=3). *P<0.01 vs NT siRNA normoxia, **P<0.01 vs NT siRNA H/R, †*P*<0.01 vs NT siRNA PM+H/R. One way ANOVA was performed using GraphPad-Prism software (version 8).

A recent study has shown increased expression of GPx and catalase in 2-day old mice heart specifically overexpressing ErbB2 in cardiomyocyte.^26^ Since cardiomyocytes from adult mice do not express ErbB2, we reasoned that endothelial ErbB2 in I/R might play a major role in RIPC-dependent protection via upregulation of antioxidant proteins. We evaluated the effect of I/R-mediated endothelial ErbB2 degradation in the expression of antioxidant proteins. As shown in Figure 4A, the expression of ErbB2 and Trx2 was significantly decreased in I/R in the heart tissue. Loss of ErbB2 and Trx2 was observed in ischemia alone without reperfusion and also during ischemia-reperfusion. However, in mice subjected to RIPC, these proteins were preserved during I/R. There was no change in the expression of SOD1, SOD2, or catalase enzymes (Figure 4A and 4B). Additionally, the depletion of ErbB2 by siRNA in HCAECs resulted in the loss of Trx2, but not SOD1, SOD2, or catalase (Figure 4C). Conversely, overexpression of ErbB2 in endothelial cells increased the expression of Trx2 (Figure 4D), demonstrating a critical role of ErbB2 in the regulation and rescue of Trx2 in RIPC. We determined whether H/R blocks the expression of ErbB2 or Trx2 using RT-PCR analysis. H/R did not cause any change in ErbB2 or Trx2 mRNA levels (Figure 4E), indicating protein degradation by H/R. To understand how Trx2 is degraded due to loss of ErbB2 in H/R, HCAECs were pretreated with autophagy, proteasomal and lysosomal inhibitors and then exposed to H/R. As shown in Figure 4F, 3-MA, and chloroquine blocked H/R-induced degradation of Trx2, demonstrating loss of Trx2 occurs through autophagy. We evaluated the role of beclin 1 and Ambra1 in Trx2 degradation, as these proteins are the master regulator of macroautophagy.^56^ As shown in Figure 4G, depletion of beclin 1 and Ambra1 resulted in partial blockade of Trx2 degradation by loss of ErbB2, indicating the involvement of both beclin 1 and Ambra1 in Trx2 autophagy. Since ATG5 is involved in the formation of autophagosomes from mitochondria^57^ and reported to be involved in selective degradation of mitochondrial proteins in yeast,^58^ we tested if ATG5 plays any role in H/R-induced degradation of Trx2. Depletion of ATG5 by siRNA in HCAECs blocked H/R-induced loss of Trx2. Interestingly, ATG5 depletion also prevented ErbB2 loss-mediated degradation of Trx2 (Figure 4H). ATG5 coimmunoprecipitated with ErbB2 and its association with ErbB2 was negatively affected by H/R (Figure 4I). We confirmed the association of ErbB2 and ATG5 in situ by PLA assay (Figure 4J). As detected by coimmunoprecipitation, PLA also showed loss of ErbB2 and ATG5 interaction in H/R-treated HCAECs (Figure 4J and 4K). Because ErbB2 is a receptor tyrosine kinase, we tested whether ErbB2 association with ATG5 results in tyrosine phosphorylation of the latter. As shown in Figure 4L, ATG5 was highly phosphorylated in normoxia and H/R induced a significant loss of tyrosine phosphorylation of ATG5 (Figure 4L). If Trx2 was required for RIPC-mediated protection of myocardial apoptosis in I/R then depletion of Trx2 should exacerbate myocardial apoptosis in I/R. We depleted Trx2 in cultured HCAECs and determined apoptosis in H/R. As shown in Figure 4M and Figure IV in the Data Supplement, loss of Trx2 accentuated apoptosis in H/R in these cells. Furthermore, treatment of Trx2-depleted HCAECs with PM treatment did not provide significant protection against apoptosis in H/R. These experiments show that Trx2 is required for the protective effect of RIPC in I/R.

### Deletion of Endothelial ErbB2 Leads to Loss of RIPC-Mediated Protection of Myocardial Perfusion in Mice

**Figure 5.**
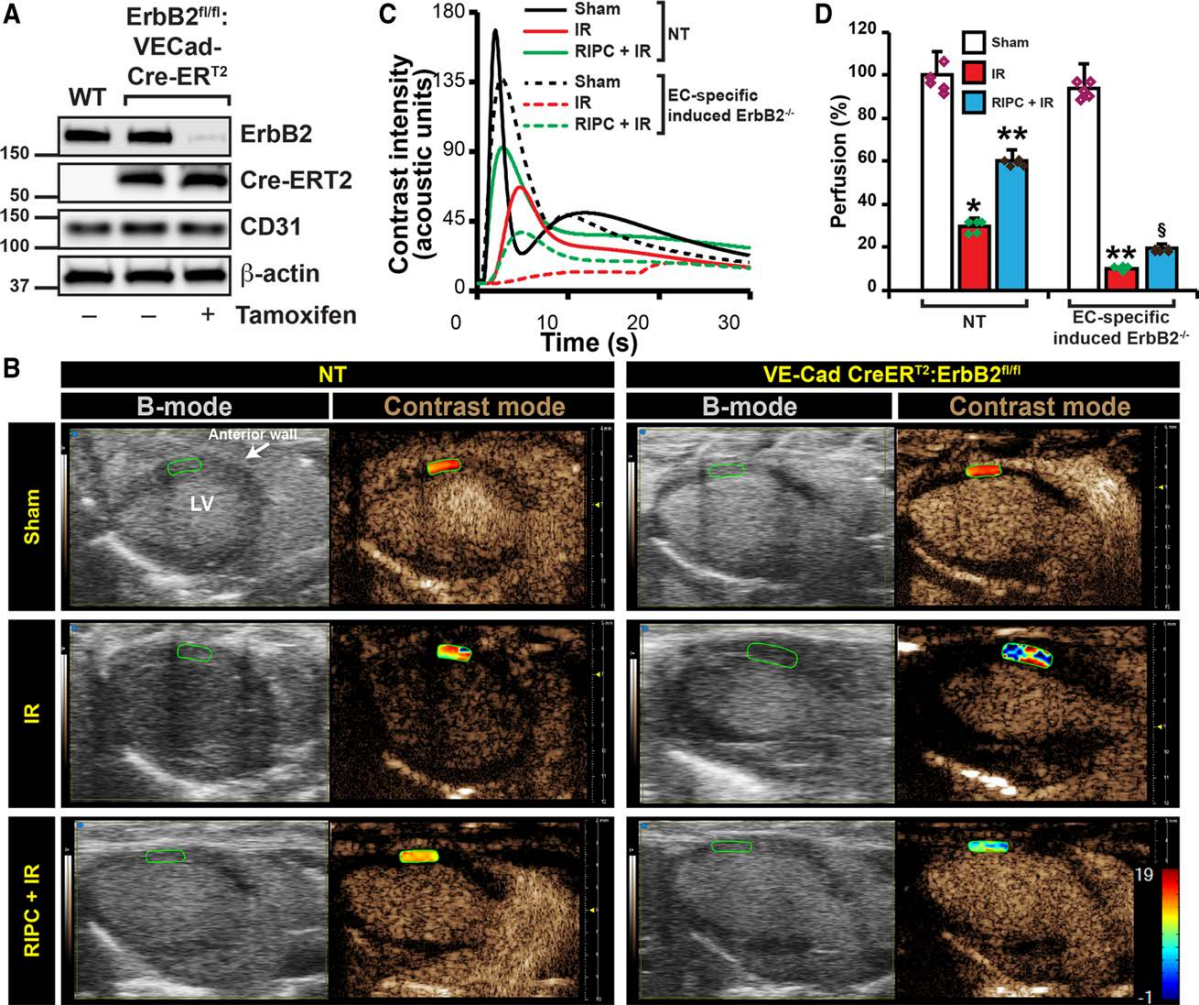
**Endothelial-specific deletion of ErbB2 (Erb-B2 receptor tyrosine kinase 2) results in loss of remote ischemic preconditioning (RIPC)-mediated protection of cardiac perfusion. A**, To generate a mouse model of endothelial-specific inducible deletion of ErbB2, we crossed ErbB2 floxed mice with VECad-Cre-ER^T2^ mice. To test the endothelial-specific deletion of ErbB2, we isolated endothelial cells from WT and ErbB2^fl/fl^:VECad-Cre-ER^T2^ mice and treated with tamoxifen (30 µg/mL) for 48 h. After the treatment, cells were lysed, and an equal amount of protein from cell lysates was analyzed for ErbB2 levels by Western blotting using anti-ErbB2 antibodies. Blots were reprobed with anti-Cre, anti-CD31, and anti-β-actin antibodies. **B**, To induce deletion of ErbB2 in endothelial cells, ErbB2^fl/fl^:VECad-Cre-ER^T2^ mice were injected with three doses of tamoxifen (75 mg/kg). After the induced deletion, ErbB2^fl/fl^:VECad-Cre-ER^T2^ and nontarget (NT) mice were subjected to sham or myocardial ischemia/reperfusion (I/R) or RIPC+I/R surgery. At the end of the reperfusion period, the nontargeted contrast agent was injected into mice via the left femoral vein while collecting B-mode/contrast mode images of the heart from the long-axis view using Vevo 3100. **C**, Contrast intensities in the left ventricle (LV) anterior wall downstream to the ligation site were quantitated and plotted over time. **D**, From the rate of contrast agent intensity change, percentage of perfusion was calculated based on the perfusion index assuming its level in the sham group is 100% and blotted as a bar graph (n=5) and shown as a heat map over the anterior LV in **A**. Values are means±SEM (NT, n=5; ErbB2^fl/fl^:VECad-Cre-ER^T2^, n=3). *P<0.01 vs NT sham, **P<0.01 vs NT I/R, §*P*<0.01 vs NT RIPC+I/R (ANOVA).

ErbB2 is an essential molecule for the vascular and neuronal development, and its deletion is embryonically lethal in mice.^59^ Therefore, we generated endothelial-specific inducible ErbB2 knockout mice (EC-ErbB2^−/−^) mice, by cross-breeding ErbB2 floxed mice^34^ with *VECad-Cre-ER*^*T2*35^ strain. When induced with tamoxifen, ErbB2 levels in endothelial cells from *ErbB2*^*fl/fl*^:*VECad-Cre-ER*^*T2*^ mice showed no detectable level of ErbB2 (Figure 5A). After inducing ErbB2 deletion in endothelial cells by tamoxifen injections, *EC-ErbB2*^−/−^ and *NT* mice were subjected to sham, I/R, and RIPC followed by I/R and myocardial perfusion was determined by contrast echocardiography. RIPC-subjected *NT* mice showed 30% increase in myocardial perfusion compared with I/R only group (Figure 5 through 5D). Endothelial-specific deletion of ErbB2 resulted in severe loss of myocardial perfusion in I/R (Figure 5B) with a large shift of perfusion peak (Figure 5C) to the right demonstrating severe vascular dysfunction. Treatment of this strain to RIPC before I/R resulted in very marginal and statistically insignificant recovery of perfusion (Figure 5D). It is important to note that 60% of total number of *EC-ErbB2*^−/−^ mice subjected to I/R died when LCA was occluded and before reperfusion. The above calculations were made only from mice that survived after reperfusion until perfusion measurements were made. These results show that endothelial-ErbB2 is essential for myocardial perfusion, loss of which causes severe vascular dysfunction resulting in significant apoptosis (Figure 3A and 3I).

### Endothelial-ErbB2 Recruits Src to Mediate eNOS Activation

**Figure 6.**
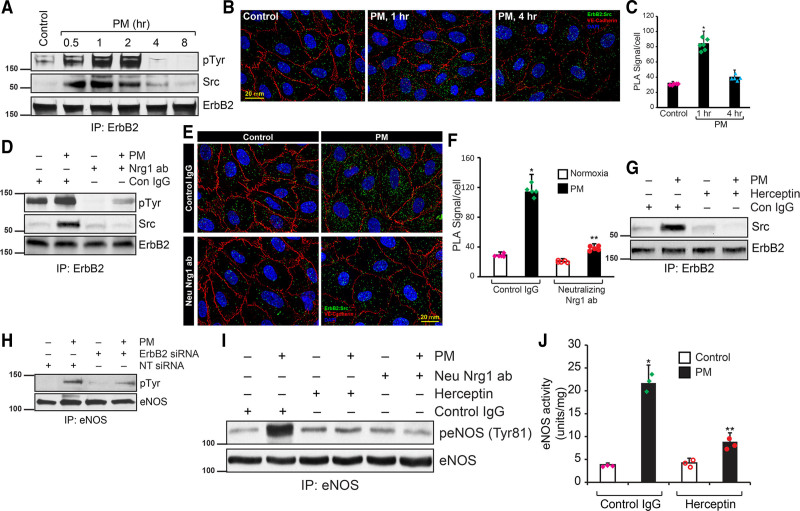
**Endothelial-ErbB2 (Erb-B2 receptor tyrosine kinase 2) directly associates with Src and activate eNOS (endothelial nitric oxide synthase). A**, Human coronary artery endothelial cells (HCAECs) were treated with preconditioned medium (PM) for the indicated period, and cell lysates were prepared. An equal amount of protein from cell lysates was immunoprecipitated using the anti-ErbB2 antibody. The immunoprecipitates were analyzed by Western blotting for tyrosine phosphorylation of ErbB2, level of associated Src, ErbB4, and ErbB2 using their specific antibodies. **B**, HCAECs were treated with CM for 1 or 4 h, and proximity ligation assay (PLA) was performed using anti-ErbB2 and anti-Src antibodies. Scale bar=20 mm. **C**, PLA signals were counted and plotted as a bar graph (n=5). *P<0.01 vs control (ANOVA). **D**, To determine the role of Nrg1 (neuregulin 1) released by HMVEC in PM, PM was pretreated with neutralizing anti-Nrg1 and then incubated with HCAECs for 1 h. At the end of treatment, cell lysates were prepared and analyzed for ErbB2 tyrosine phosphorylation and its interaction with Src immunoprecipitation followed by Western blotting. **E**, HCAECs were incubated with PM or Nrg1 neutralized-PM for 1 h. and PLA was performed using anti-ErbB2 and anti-Src antibodies. Scale bar=20 mm. **F**, PLA signals were counted and plotted as a bar graph (n=5). *P<0.01 vs control (ANOVA). **G**, HCAECs were pretreated with Herceptin and incubated with PM for 1 h, and cell lysates were prepared. An equal amount of proteins from cell lysates were immunoprecipitated with anti-ErbB2 antibodies, and the immunocomplexes were analyzed for associated Src by Western blotting. **H**, HCAEC were transfected with ErbB2 siRNA, treated with PM for 1 h, and cell lysates were prepared and analyzed for eNOS tyrosine phosphorylation. **I**, HCAECs were pretreated with control IgG, Herceptin, or Nrg1 neutralizing antibodies and then incubated with PM for 1 h, and cell lysates were prepared. An equal amount of proteins from cell lysates were analyzed for eNOS-Tyr81 phosphorylation by Western blotting using its specific antibodies. **J**, HCAECs were pretreated with control IgG or Herceptin and incubated with PM for 1 h, and cell lysates were prepared. An equal amount of protein from cell lysates was analyzed for eNOS activity and plotted as a bar graph. *P<0.01 vs control IgG, **P<0.01 vs control IgG+PM (ANOVA).

The interaction of endothelial-secreted Nrg1 with its receptor ErbB2 expressed in myocytes is well documented.^14,23^ However, the expression and interaction of ErbB2 in endothelial cells of myocardium remain unknown. Since Nrg1 protects against endothelial dysfunction by enhancing eNOS function and ErbB2 is a receptor for Nrg1, we speculated Nrg1-ErbB2 interaction in the coronary artery endothelial cells. First, we determined whether the ErbB2 receptor is expressed in the coronary endothelium. As shown in Figure 6A (bottom panel), a robust expression of ErbB2 was detected in HCAEC. Additionally, ErbB2 was strongly phosphorylated in HCAECs when treated with PM, and the phosphorylation was peaked at 2 hours of treatment. Subsequent analysis of the immunoprecipitate showed that ErbB2 is associated with Src in response to treatment with PM (Figure 6A). We further confirmed the recruitment of Src by ErbB2 using an in situ PLA assay with control and PM-treated HCAECs. As shown in Figure 6B and 6C, in response to PM treatment, significant PLA signals were obtained, demonstrating the recruitment of Src by ErbB2. We determined whether Nrg1 in PM would interact with ErbB2 and would subsequently recruit Src. As shown in Figure 6D, neutralizing anti-Nrg1 antibody blocked ErbB2 tyrosine phosphorylation, as well as recruitment of Src. Additionally, in situ PLA showed that neutralizing Nrg1 pretreatment of PM abolished interaction of ErbB2 with Src (Figure 6E and 6F). To confirm the role of ErbB2 in PM-induced Src activation, we used herceptin to block ErbB2 function and evaluated its interaction with Src. Blockade of ErbB2 function by herceptin abrogated PM-induced Src interaction with ErbB2 (Figure 6G). A study has shown that Src kinase phosphorylates eNOS directly at Tyr 83 (equivalent to Tyr 81 in humans) in bovine aortic endothelial cells and enhances its basal activity.^30^ Since we found strong tyrosine phosphorylation in eNOS, we determined whether Src kinase activates eNOS in HCAEC in response PM treatment. PM caused acute activation of Src kinase as detected by phosphorylation at Tyr 416 (Figure VA in the Data Supplement). This activation was sensitive to the neutralization of Nrg1 in PM when the Nrg1 neutralizing antibody was added to PM (Figure VB in the Data Supplement). Either depletion of ErbB2 by its siRNA or blockade of its function by herceptin attenuated PM-induced Src activation (Figure VC and VD in the Data Supplement). Next, we tested the role of ErbB2 in PM-induced eNOS activation and its enzyme activity. As shown in Figure 6H, loss of ErbB2 reduced tyrosine phosphorylation of eNOS by PM. To understand the role of Nrg1 and ErbB2 in PM-induced eNOS activation, we neutralized Nrg1 and ErbB2 using neutralizing-Nrg1 antibody and herceptin, respectively, and evaluated eNOS-Tyr81 phosphorylation. Treating HCAECs with PM resulted in several-fold induction of eNOS-Tyr81 phosphorylation (Figure 6I). However, both herceptin and neutralizing Nrg1 antibodies blocked PM-induced eNOS-Tyr81 phosphorylation (Figure 6I). Herceptin also decreased PM-induced eNOS enzyme activity to basal levels (Figure 6J). Collectively, these experiments provide compelling evidence for the role of endothelial ErbB2-dependent Src-mediated eNOS activation in H/R and also suggest a critical role of Nrg1 in RIPC-induced activation of eNOS and NO release.

### Endothelial ErbB2/ErbB4 Heterodimers Recruit Src, Mediate eNOS Activation, and NO Release

**Figure 7.**
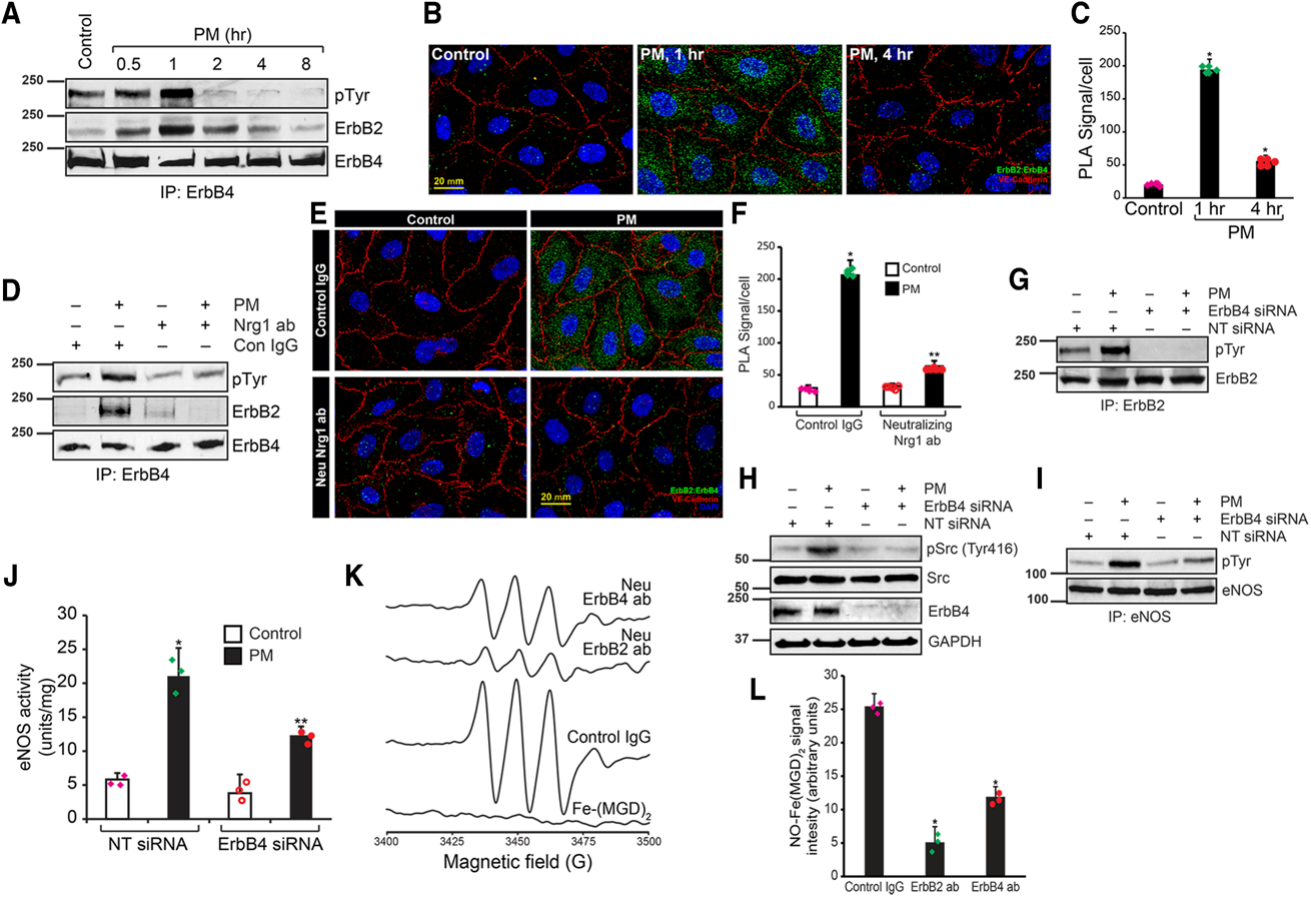
**Endothelial ErbB2 (Erb-B2 receptor tyrosine kinase 2) dimerizes with ErbB4, recruits Src and mediates Nrg1 (neuregulin 1)-dependent eNOS (endothelial nitric oxide synthase) activation during ischemic preconditioning. A**, HCAECs were treated with preconditioned medium (PM) for the indicated period, and cell lysates were prepared. An equal amount of protein from cell lysates was immunoprecipitated using anti-ErbB4 antibody, and the immunoprecipitates were analyzed by Western blotting for tyrosine phosphorylation of ErbB4 and associated ErbB2 levels using their specific antibodies. **B**, To study the in vivo interaction of ErbB2 and ErbB4, HCAECs were treated with PM for 1 or 4 h, and proximity ligation assay (PLA) was performed using anti-ErbB2 and anti-ErbB4 antibodies. Scale bar=20 mm. **C**, Green foci-proximity signals of ErbB2 and ErbB2 association were counted and plotted as a bar graph. *P<0.01 vs control (ANOVA). **D–F**, HCAECs were incubated with PM or Nrg1 neutralized-PM for 1 h, and either cell lysates were prepared and analyzed for ErbB4 tyrosine phosphorylation and its interaction with ErbB2 by Western blotting as described in **A** or subjected to PLA (**E**) using anti-ErbB2 and anti-ErbB4 antibodies as described in **B**. PLA signals were counted and plotted as a bar graph (n=5), Scale bar=20 mm (**F**). *P<0.01 vs control IgG; **P<0.01 vs neutralizing anti-Nrg1 antibody+PM. **G–J**, HCAECs were transfected with ErbB4 siRNA, treated with PM for 1 h., and the cell lysates were prepared and analyzed for ErbB2 tyrosine phosphorylation (**G**), Src activation (**H**), or eNOS tyrosine phosphorylation (**I**). An equal amount of protein from cell lysates was analyzed for eNOS activity (**J**). *P<0.01 vs nontarget (NT) siRNA; **P<0.01 vs NT siRNA+PM (ANOVA). **K**, To study the role of ErbB2 and ErbB4 in NO formation in coronary arteries, left coronary artery were isolated and incubated with control IgG, neutralizing anti-ErbB2 or neutralizing anti-ErbB4 antibodies, and NO release in response to ACh (10 µmol/L) was determined by electron spin resonance (EPR) using Fe-(MGD)_2_. **L**, Spin count of the NO-Fe(MGD)_2_ was calculated from EPR signals were plotted as a bar graph. *P<0.01 vs control IgG (ANOVA).

ErbB2 does not directly interact with its ligand, and its ligand-depended activation requires heterodimerization with other ErbB members.^60,61^ As shown in Figure 3C and 3G, ErbB4 levels were unaffected by I/R in myocardium and H/R in HCAECs. In addition, ErbB family of receptors are activated by phosphorylation of their multiple tyrosine residues.^62^ Therefore, we determined whether ErbB2 dimerizes with ErbB4 in response to RIPC and is activated by Nrg1. We immunoprecipitated ErbB4 and probed with antiphospho-tyrosine antibody. As shown in Figure 7A, ErbB4 was strongly phosphorylated in HCAECs when treated with PM and phosphorylation peaked at 1-hour period of treatment. Subsequent analysis of the immunoprecipitate showed that ErbB2 was associated ErbB4 in response to treatment with PM (Figure 7A). We further confirmed ErbB2/ErbB4 heterodimer in response to PM treatment by PLA. As shown Figure 7B and 7C, ErbB2 and ErbB4 dimerization peaked at 1 hour of treatment with PM. Neutralization of Nrg1 in PM abrogated PM-induced ErbB4 tyrosine phosphorylation (Figure 7D) and its association with ErbB2 (Figure 7E and 7F). These results show that Nrg1 in the PM is essential for ErbB4-mediated ErbB2 activation and propagation of the signals downstream. To study the role of ErbB4 in Src and eNOS activation, we depleted ErbB4 in HCAECs using siRNA. Depletion of ErbB4 by siRNA in HCAECs completely blocked PM-induced ErbB2 tyrosine phosphorylation (Figure 7G), Src activation (Figure 7H), eNOS tyrosine phosphorylation (Figure 7I), and its activity (Figure 7J). Additionally, neutralization of either ErbB2 or ErbB4 caused the loss of NO formation in LCA (Figure 7K and 7L). Further, inhibition of Src activity by PP2 abrogated PM-induced eNOS tyrosine phosphorylation (Figure VIA in the Data Supplement). Likewise, blocking Src kinase activity by dnSrc blocked eNOS activating tyrosine phosphorylation (Figure VIB through VID in the Data Supplement) as well as its enzymatic activity (Figure VIE in the Data Supplement). These results together shed light on the functions of ErbB receptors in endothelial cells of coronary arteries and demonstrate that ErbB2/ErbB4 heterodimers mediate RIPC-induced eNOS activation.

### Nrg1β Induces eNOS via Heterodimerized Endothelial ErbB2/ErbB4-Mediated Src Activation

Hypoxia and subsequent reoxygenation can induce HMVECs to secrete several factors into the medium. To evaluate if Nrg1 released into the medium specifically activates ErbB2/ErbB4-Src-eNOS signaling in HCAECs, we treated HCAECs with recombinant protein corresponding to human Nrg1β1 activating domain and determined eNOS activity. As shown in Figure VIIA in the Data Supplement, hrNrg1β induced eNOS activity by >10-folds at 30 minutes of treatment. Induction of eNOS activity by hrNrg1β at 30 minutes treatment correlated with the magnitude of ErbB2 and ErbB4 tyrosine phosphorylation (Figure VIIA through VIIC in the Data Supplement), Src activation (Figure VIID in the Data Supplement), and eNOS tyrosine phosphorylation (Figure VIIE in the Data Supplement). Blockade of ErbB2 and ErbB4 functions by herceptin and ErbB4 neutralizing antibody,^63^ respectively, significantly reduced rhNrg1β1-induced ErbB2, ErbB4, eNOS tyrosine phosphorylation, and Src activation (Figure VIIB through VIIE in the Data Supplement). Additionally, overexpression of dnSrc in HCAECs blocked rhNrg1β1-induced eNOS tyrosine phosphorylation as well as it activity (Figure VIIG and VIIH in the Data Supplement). Collectively, these results demonstrate that Nrg1β stimulates eNOS activity by tyrosine phosphorylation via ErbB2/ErbB4-Src signaling.

## Discussion

In this report, we have identified Nrg1β as a RIPC factor and provide evidence that RIPC induced Nrg1β is secreted by microvascular endothelial cells of gastrocnemius muscle, which interacts with endothelial ErbB2 preventing its degradation in I/R resulting in inhibitory phosphorylation of ATG5 and consequent protection of Trx2 degradation in IR providing a significant protection against MI. We have shown that enhanced levels of O_2_^•−^ are produced in the gastrocnemius muscle and the level of endothelial Nrg1β expression is also increased due to RIPC. When PEG-SOD was applied, the levels of O_2_^•−^ was decreased with concomitant reduction in Nrg1β levels, indicating that O_2_^•−^ produced in RIPC induces Nrg1β expression. Additionally, we found that ErbB2 is expressed in the endothelial cells, but not in cardiomyocytes of adult mice heart, which is degraded by I/R. Degradation of endothelial ErbB2 results in autophagy of mitochondrial Trx2 that is required for protection against cardiomyopathy in I/R. ErbB2 constitutively retains ATG5 in an inactivated tyrosine-phosphorylated state, which is dephosphorylated in H/R due to loss of ErbB2 resulting in activation of ATG5 that selectively degrades Trx2. Our study identified 2 complementary pathways activated by RIPC-mediated Nrg1 secretion: (1) ErbB2/4-cSrc-NO axis and (2) ErbB2/4-ATG5-Trx2 axis that converge to protect myocardial perfusion and infarction in I/R (Figure VIII in the Data Supplement). Both vascular and cardiac mechanisms are involved in RIPC-mediated protection of I/R due to inhibition of ErbB2 degradation by Nrg1 secreted in RIPC. The ErbB2/4-cSrc-NO axis is critically important in restoration of vascular function and myocardial perfusion, whereas ErbB2/4-ATG5-Trx2 pathway that protects myocardial apoptosis due to upregulation of Trx2 loss, which is known to result in massive myocardial apoptosis.^28,29^

RIPC was first demonstrated to protect the myocardium from I/R injury in dogs, and since then cardioprotective benefits of RIPC has been verified in mice, rats, rabbits, pigs, and primates.^64,65^ RIPC is an effective, low-cost, nonpharmacological, and noninvasive therapy to protect the heart against I/R-induced MI.^2,6^ However, translation of RIPC to clinics as a cardioprotective intervention did not take effect yet due to inconclusive outcomes from human clinical trials. A meta-analysis 27 clinical trials documented that RIPC shortened the hospital stay period and reduced MI.^66^ However, large randomized multi-center studies could not establish the benefits of RIPC with respect to MI, stroke, troponin release, and the extend of hospital stay.^67^ A comprehensive examination of several clinical trials that tested RIPC reveals disparities in the method of RIPC, duration, choice, and size of the vascular bed used for RIPC. In addition, timing from RIPC to surgery, the type of anesthesia used and more importantly heterogeneity, as well as the occurrence of comorbidities among the study subjects could have impacted the study outcome. Further, age and comorbidities, such as diabetes and dyslipidemia, are the prime factors in patients that could affect the RIPC outcome adversely. Since specific protective factor (s; or even whether it is humoral, neural, or other) released by RIPC has not been identified,^2^ it remains challenging to determine whether age or comorbidities affect the generation of RIPC factors.

Trx2 knock out mice die in utero with massive myocardial apoptosis, demonstrating the critical requirement of Trx2 in the protection against myocardial apoptosis.^29^ Our findings of the regulation of Trx2 protein levels by endothelial ErbB2-dependent inactivation of ATG5 by tyrosine phosphorylation provides a mechanism by which endothelial ErbB2 regulates cardiac antioxidant system, as Trx2 is located in the mitochondria. Cardiomyocytes make up 70% to 85% of heart by volume, but endothelial cells are the most numerous and constitute up to 64% by number in the mammalian heart.^55^ Considering the total volume of mitochondria in cardiomyocytes, Trx2 loss detected in infarcted myocardium (Figure 4A) may be from both endothelial cells and cardiomyocytes. Since adult mouse cardiomyocytes did not express any detectable amount of ErbB2 (Figure 3E), it is possible that NO generated by endothelial cells inhibits loss of Trx2 by autophagy in cardiomyocytes as it is known that NO impairs autophagy.^68^ Several reports provided evidence that inhibition of receptor tyrosine kinases activate autophagy.^69,70^ Further, our data in Figure 4G show that although MG-132 impairs autophagic flux, the expression of Trx2 was decreased. MG132 has been shown to compromise autophagosome formation and thereby stalling autophagy flux in silkworm Bombyx mori ovarian cell line.^71^ However, MG132 is shown to have opposite effect in human cells.^72^ Interestingly, inhibition of proteosome by PS341 or MG132 induce degradation of ubiquitin proteosome system clients by autophagy.^72,73^ However, we did not observe any significant change in LC3-II levels in MG132 treatment compared with controls. Therefore, we assume that MG132 neither altered autophagy flux in endothelial cells used in our study nor interfered with H/R-mediated degradation of Trx2. Trx2 deletion in the myocytes results in the activation of ASK1 and promotes myocardial apoptosis.^29^ Therefore, degradation of Trx2 in I/R could promote ASK1-mediated apoptosis of cardiac tissue. The rescue of I/R -mediated loss of Trx2 by RIPC via Nrg1β-dependent protection of loss of endothelial ErbB2 in mice is an important previously unrecognized mechanism of RIPC-mediated protection against MI. The presence of ErbB2 and its role in angiogenesis in the venous endothelium was first reported by Russell et al.^74^ A recent report^75^ has demonstrated the presence of ErbB2 in the venous endothelium confirming the report of Russell et al.^74^ Our data show a specific role of endothelial ErbB2/ErbB4 in eNOS activation via Src in I/R, contrary to established role of myocyte ErbB2/ErbB4 in I/R mediated survival signaling. Thus, there is a clear distinction regarding the protective mechanism in RIPC-mediated Nrg1β versus paracrine interaction of microvascular endothelium-derived Nrg1β with myocyte ErBb2/ErbB4. Perfusion of myocardium distal to the site of occlusion is the AAR for infarction, as coronary arteries are functional end arteries.^4,76^ Therefore, within a given AAR, both the duration and severity of reduction in blood flow determine the nature and amount of injury,^4,76^ underscoring the importance of coronary artery dysfunction in MI. Loss of NO release by endothelial cells in I/R is one of major mechanism in MI development. Our study also provides direct evidence that RIPC enhanced eNOS activity and improved of cardiac perfusion. Therefore, protection of endothelial function due to coronary artery preconditioning could be a major mechanism in RIPC that reduces MI in I/R. Consistent with this notion, when Nrg1 neutralizing antibody was injected into mice before RIPC of FA, the protective effect of RIPC in endothelial dysfunction of the coronary arteries was abrogated concomitant with loss of a protective effect against MI. These data show that the correction of endothelial dysfunction of the coronary arteries is pivotal in extending the beneficial effect of RIPC to the myocardium.

Despite the documented beneficial role of Nrgs in cardiac function, circulating Nrg1 level was associated with heart failure severity,^11^ which did not correlate with the ability to repair damaged myocardium.^77^ In this regard, our study made an important finding that administration of rhNrg1 before ischemic insult protects myocardium while introduction of rhNrg1 postischemic stress aggravated MI. Ischemic stress results in loss of ErbB2 level and RIPC preserves endothelial ErbB2 which is essential to mediate Nrg1β signaling in repair and recovery of damaged myocardium. It is important to note that unlike EGFR (ErbB1) homodimers, ErbB2/ErbB4 heterodimers avoid lysosomal degradation^60^ and when activated by Nrg1, their stability and signaling potency is further enhanced.^78^ Nrg1 signaling in adult heart is mediated by ErbB2/ErbB4 heterodimers^23^ which modulate cardiomyocyte contractile functions.^22^ A previous study has shown that Nrg1β is secreted in conditions of oxidative stress in the cardiac microvascular endothelial cells.^79^ Since short periods of I/R in a remote organ is expected to generate ROS, Nrg1β secretion could have been stimulated by ROS. Further, proteolytic processing of Nrg1β could also have been activated due to ROS, and H_2_O_2_ has been shown to induce Nrg1β.^79^ We have demonstrated that conditioned media from HMVEC exposed to H/R activates ErbB2 in the HCAECs. This study recapitulates the possible in vivo secretion of Nrg1β to the circulation and its interaction at the target site of coronary endothelium. Therefore, Nrg1β secretion could be correlated with the humoral theory of RIPC where the humoral factor is <30 kDa as proteolytic cleavage of 115 kDa Nrg1β produces the 25 kDa active Nrg1β. Further, either herceptin or knockdown of ErbB2 in the endothelium abrogates RIPC protective effect suggesting that secreted Nrg1β interaction with endothelial ErbB2 is required for protective action of RIPC. In addition to protection against endothelial dysfunction, RIPC of FA protected against MI, the specific role of remotely released Nrg1β is established in MI, as neutralizing antibody to Nrg1 abrogated the protective effect of RIPC. Our study does not exclude the possibility that additional RIPC factors such as erythropoietin^80^ and nitrite^81^ play a significant role in protective mechanism of RIPC.^82^ It is interesting to note that many of these factors are downstream effector of Nrg1.

## Acknowledgments

V. Kundumani-Sridharan and K.C. Das participated in conception and design of the study. V. Kundumani-Sridharan, J. Subramani, and C. Owens performed experiments and collection of data. V. Kundumani-Sridharan and J. Subramani participated in data analysis. V. Kundumani-Sridharan, J. Subramani, and K.C. Das participated in interpretation of data and discussion. V. Kundumani-Sridharan and K.C. Das participated in article preparation.

## Sources of Funding

The work is supported by National Heart Lung and Blood Institute of National Institutes of Health grant number HL144610, HL 132953, HL 107885 to K.C. Das.

## Disclosures

None.

## Supplementary Material


