# Supplementary material for: Nrg1β Released in Remote Ischemic Preconditioning Improves Myocardial Perfusion and Decreases Ischemia/Reperfusion Injury via ErbB2-Mediated Rescue of Endothelial Nitric Oxide Synthase and Abrogation of Trx2 Autophagy
Source: Arterioscler Thromb Vasc Biol. 2021 May 27;41(8):2293–314. doi: 10.1161/ATVBAHA.121.315957 (PMC8288485; doi:10.1161/ATVBAHA.121.315957)
Supplement: Supplementary file 1 [file atv-41-2293-s001.pdf]

SUPPLEMENTAL MATERIALS

Nrg1β released in RIPC improves myocardial perfusion and decreases I/R injury via ErbB2-mediated rescue of eNOS and abrogation of Trx2 autophagy

Running Title: Nrg1β protects MI via rescue of Trx2 degradation

Venkatesh Kundumani-Sridharan[1], Jaganathan Subramani[1], Cade Owens[1], and Kumuda C. Das[1*]

*[1]Department of Internal Medicine, Texas Tech University Health Sciences Center, 3601 4th Street, Lubbock TX79430.

*Address for Correspondence:
Kumuda C. Das, Ph.D
Department of Internal Medicine
Texas Tech University Health Sciences Center
3601 4th Street, MS6598
Lubbock, TX 79424
Phone: (806) 743-6747
E-mail: kumuda.das@ttuhsc.edu

Major Resources Table

In order to allow validation and replication of experiments, all essential research materials listed in the Methods should be included in the Major Resources Table below. Authors are encouraged to use public repositories for protocols, data, code, and other materials and provide persistent identifiers and/or links to repositories when available. Authors may add or delete rows as needed.

Animals (in vivo studies)

Species	Vendor or Source	Background Strain	Sex	Persistent ID / URL
Mouse - *Mus musculus*	Charles River Laboratory	C57BL/6NCrl	Male	https://www.criver.com/products-services/find-model/c57bl6-mouse?region=3611

Genetically Modified Animals

	Species	Vendor or Source	Background Strain	Other Information	Persistent ID / URL
**Parent – Male**	Mouse	European Mouse Mutant Achieve	C57BL/6<br>Substrain unknown	ErbB2 fl/fl (Strain was generated from embryos in Charles River Laboratory)	EMMA ID: EM:08023
**Parent - Female**	Mouse	Charles River Laboratory	C57BL/6NCrl		
**Parent – Male**	Mouse	Dr. M. Luisa Iruela-Arispe, University of California, Los Angeles, CA	C57BL/6J	CIVE23 (C57BL/6J, Inducible, VE-cadherin Cre-recombinase, line#23	VE Cad CreERT2
**Parent - Female**	Mouse	Dr. M. Luisa Iruela-Arispe, University of California, Los Angeles, CA	C57BL/6J	CIVE23 (C57BL/6J, Inducible, VE-cadherin Cre-recombinase, line#23	VE Cad CreERT2

Antibodies

Target antigen	Vendor or Source	Catalog #	Working concentration	Lot # (preferred but not required)	Persistent ID / URL
Nrg1 antibody	R & D Systems	AF-396-NA	Neutralizing ab		
eNOS antibody	BD Biosciences	610296	1:2000 (WB)	7236996	

Nrg1 antibody	Cell Signaling Technology	2573	1:1000 (WB to detect full length Nrg1)		
ErbB2 antibody	Cell Signaling Technology	4290	1:1000 (WB)		
Phospho-eNOS (Ser1177) antibody	Cell Signaling Technology	9571	1:1000 (WB)		
Phospho-Src (Tyr416) antibody	Cell Signaling Technology	2101	1:1000 (WB)		
Ambra1 antibody	Cell Signaling Technology	24907	1:1000 (WB)		
BCLN1 antibody	Cell Signaling Technology	3495	1:1000 (WB)		
Nrg1 antibody	Santa Cruz Biotechnology	SC-28916	1:250 (IF)		
ErbB4 antibody	Santa Cruz Biotechnology	SC-283	1:1000 (WB)		
VE-cadherin antibody	Santa Cruz Biotechnology	SC-6458	1:1000 (WB)		
Trx2 antibody	Santa Cruz Biotechnology	SC-50336	1:1000 (WB)		
Heregulin antibody	Pierce Biotechnology	RB-276-P0	1:2000 (WB)		
ErbB4 antibody	Pierce Biotechnology	MA5-13016	Neutralizing ab		
ErbB4 antibody	Pierce Biotechnology	MA1-861	1:200 (PLA)		
Src antibody	Millipore	05-184	1:2000 (WB)		
Phospho-Tyr antibody	Millipore	05-321	1:200 (IP),<br>1:2500 (WB)		
CD31 antibody	Abcam	ab9498	1:1000 (WB)<br>1:200 (IF)		
ATG5 antibody	Abcam	ab108327	1:1000 (WB)		

			1:250 (IF)		
Tom20 antibody	Abcam	ab56783	1:2000 (WB)		
Her2 antibody (trastuzumab)	Absolute Antibody Ltd	Ab00103-10.0	Neutralizing ab		
Phospho-eNOS (Tyr 83) antibody	Dr. Richard C. Venema, Medical College of Georgia, GA		1:500 (WB)<br>1:100 (IF)		
Alpha-actinin antibody	Sigma	AA7732	1:5000 (WB)<br>1:500 (IF)		
GAPDH−Peroxidase antibody	Sigma	G9295	1:30,000 (WB)	112M4761V	
β-actin−Peroxidase antibody	Sigma	A3854	1:30,000 (WB)	043M4825V	

DNA/cDNA Clones

Clone Name	Sequence	Source / Repository	Persistent ID / URL
pcDNA3	https://tools.thermofisher.com/content/sfs/vectors/pcdna3_seq.txt	Invitrogen Corp	Cat. No. V79020
pcDNA3-ErbB2	http://www.addgene.org/16257/sequences/	Addgene Plasmid	Cat. No. 16257, http://www.addgene.org/16257/

Cultured Cells

Name	Vendor or Source	Sex (F, M, or unknown)	Persistent ID / URL
Human coronary artery endothelial cells	Lonza Walkersville, Inc	Male	Cat. NO. CC-2585
Human Cardiac Microvascular Endothelial Cells	Lonza Walkersville, Inc	Male	Cat. No. CC-7030

Supplementary Figures:

Fig S1

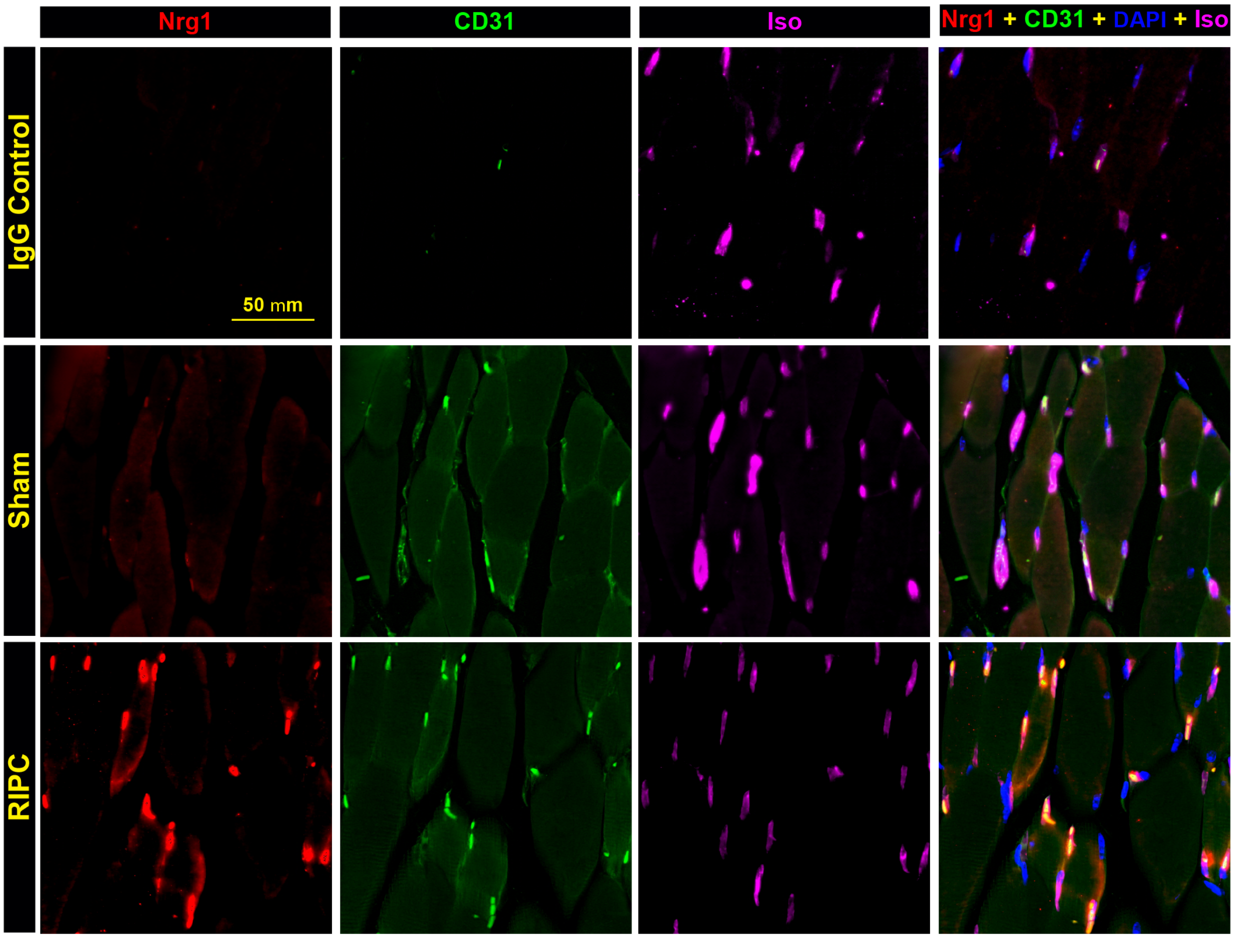

Fig S1. Magnified view of Fig 1B including separate channels and IgG control. We used IgG control for both Nrg1 and CD31 antibodies (rabbit and mouse)

Fig. S2

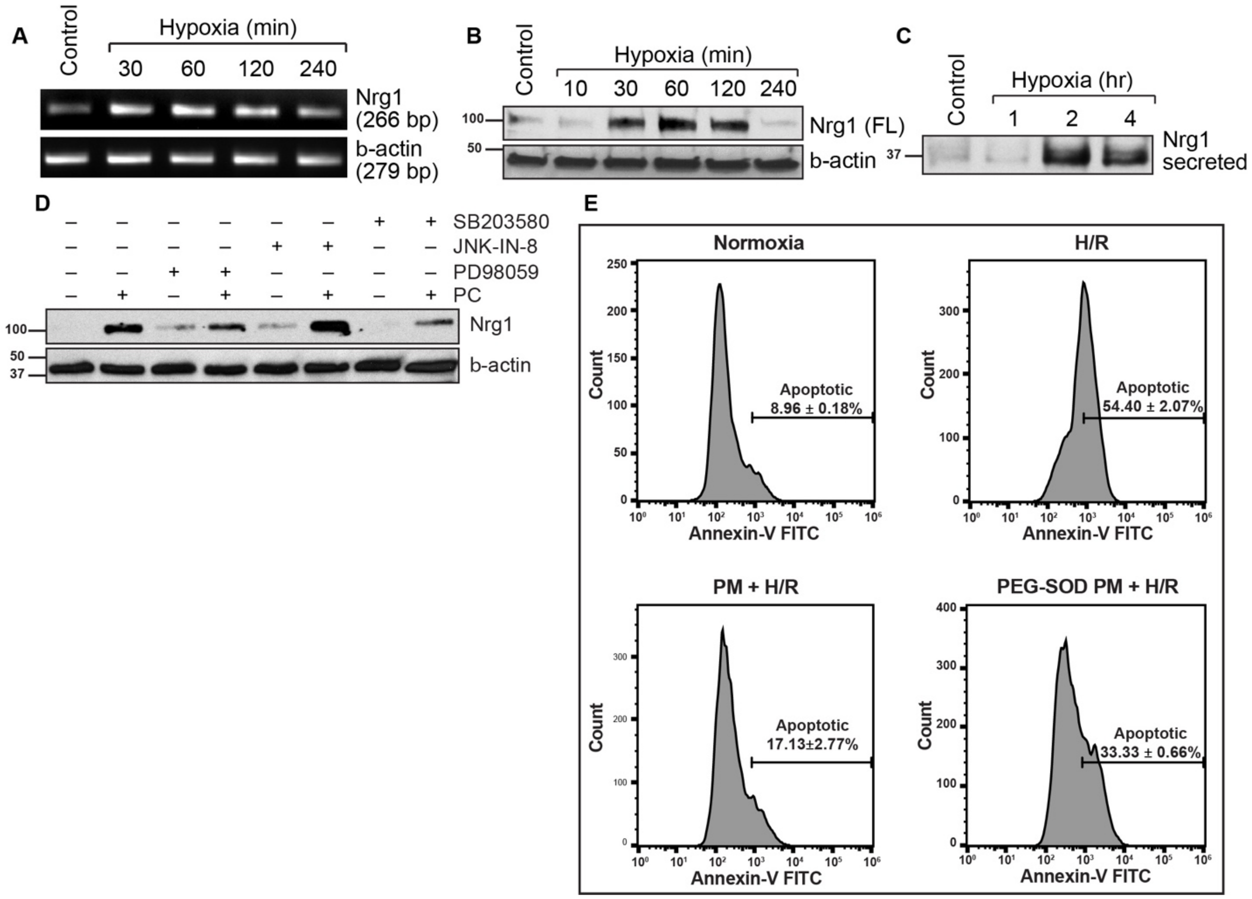

Fig. S2. Short duration-hypoxia induces Nrg1 expression and its release by microvascular endothelial cells. (A) HMVEC were exposed to hypoxia for the indicated period followed by 30 minutes of reoxygenation and RNA isolated. RT-PCR was performed to determine the expression level of Nrg1β and β -actin. **(B-C)** HMVEC were exposed to H/R as described in panel (a), and at the end of treatment, cell lysates were prepared, and the medium was collected. Nrg1 expression (B) and release level (C) were determined in cell lysates and medium, respectively, by Western blotting. **(D)** HMVEC were pretreated with PD98059, JNK-IN-8, or SB203580 and then preconditioned (1 hour hypoxia/30 minutes reoxygenation). At the end of the preconditioning, cell lysates were prepared, and Nrg1 expression levels were analyzed in cell lysates by Western blotting. **(E)** HMVECs were incubated with or without PEG-SOD for 6 hours and subjected to PC. PM was collected. HCAECs were treated with or without PM and exposed to H/R. The HCAECs undergoing apoptosis were labeled with annexin V-FITC conjugate, and the percentage of apoptotic cells was quantitated by FACS analysis using Attune NxT Flow Cytometer.

Fig S3

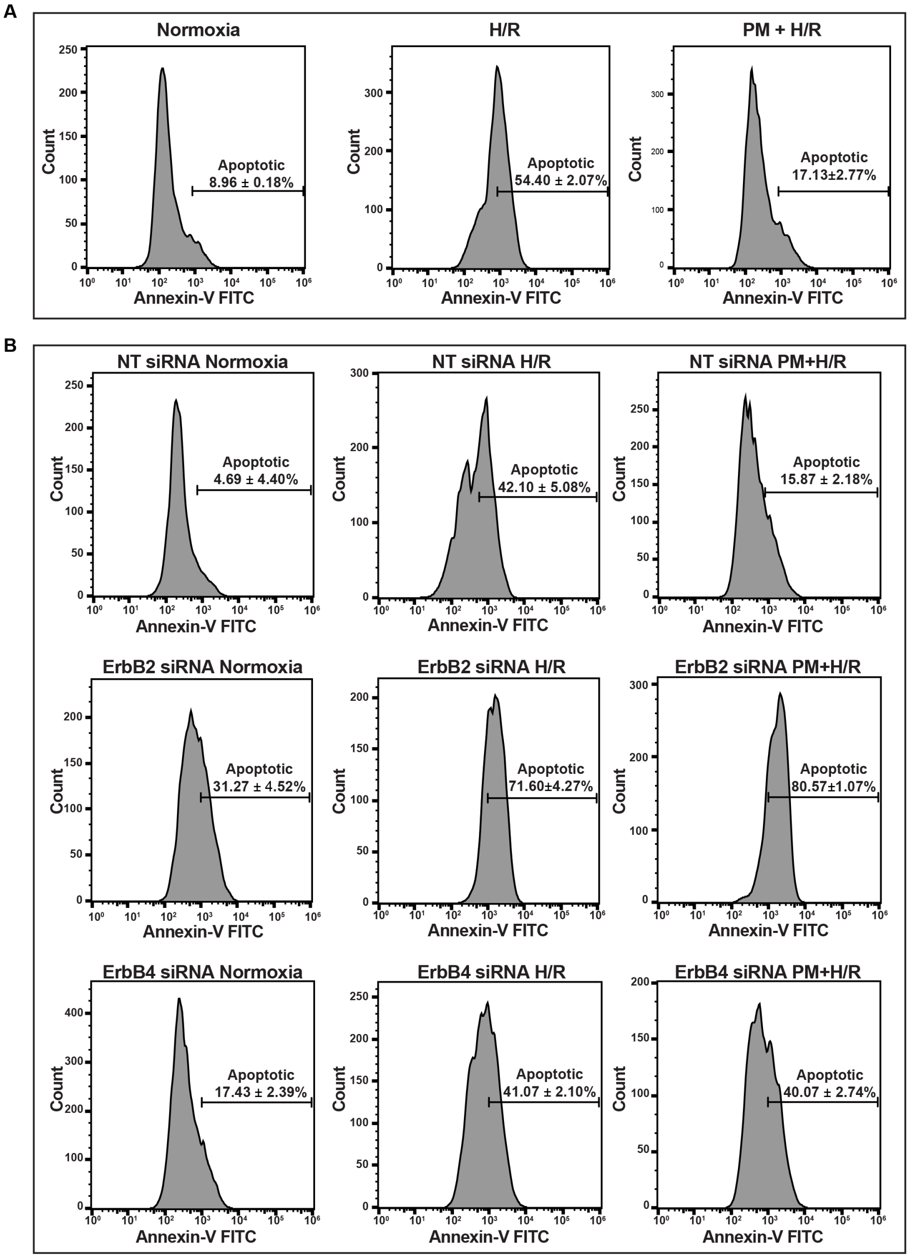

Fig. S3. Loss of ErbB2 negates RIPC-mediated protection of endothelial cell from apoptosis. **(A)** HCAECs were pretreated with or without PM and exposed to H/R. At the end of treatment, cells undergoing apoptosis were labeled with annexin V-FITC conjugate, and the percentage of apoptotic cells was quantitated by FACS analysis using Attune NxT Flow Cytometer. **(B)** HCAECs were transfected with NT or ErbB4 or ErbB4 siRNA (100 nM), and after recovery from

transfection, they were treated with or without PM and exposed to H/R. Cells apoptosis was determined as in (A).

Fig S4

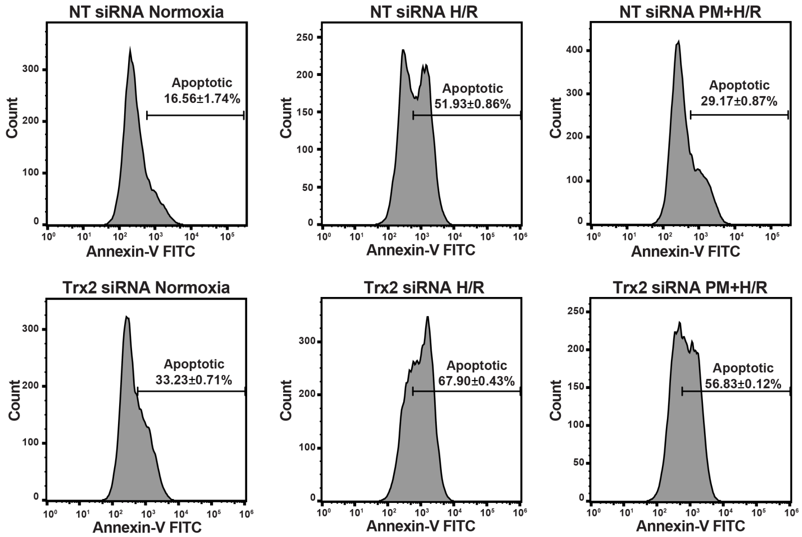

Fig. S4. Trx2 depletion abrogates RIPC-induced protection of HCAECs from apoptosis. HCAECs were transfected with NT or Trx2 siRNA (100 nM), and after recovery from transfection, they were treated with or without PM and exposed to H/R. Apoptotic cells were labeled with annexin V-FITC conjugate, and the percentage of apoptotic cells was quantified. **(C)** HCAEC were transfected with ErbB2 siRNA, treated with PM for 1 hr., and cell lysates were prepared and analyzed for Src activation.

Fig. S5

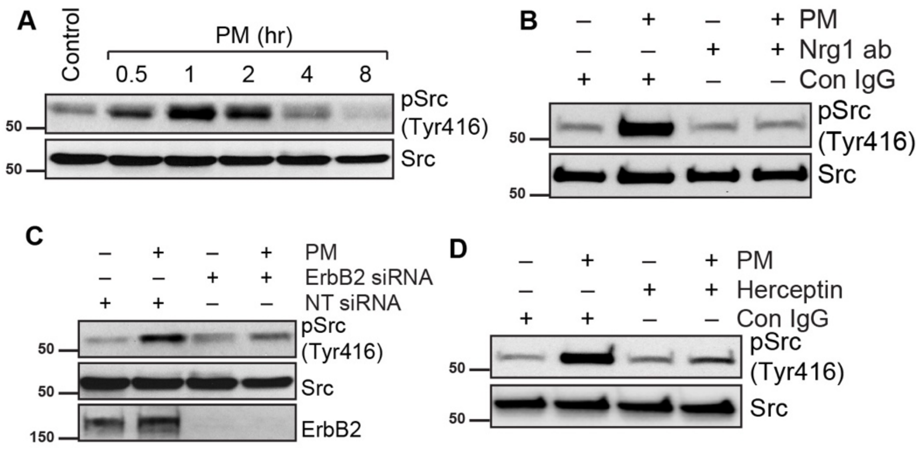

Fig. S5. PM-induced Src activation is mediated by Nrg1 and ErbB2. (A) HCAECs were treated with PM for indicated periods, lysed, and an equal amount of protein from cell lysates were analyzed by Western blotting for Src activation by using anti-phospho Src (Tyr416) antibody. **(B)** HCAECs were incubated with PM or Nrg1 neutralized PM for 1 hr., cell lysates were collected and analyzed for Src activation as described in panel (A). **(D)** HCAECs were pretreated with Herceptin and incubated with PM for 1 hr., and cell lysates were prepared. Cell lysates were analyzed for Src activation by Western blotting using anti-pSrc(Tyr416) antibodies.

Fig. S6

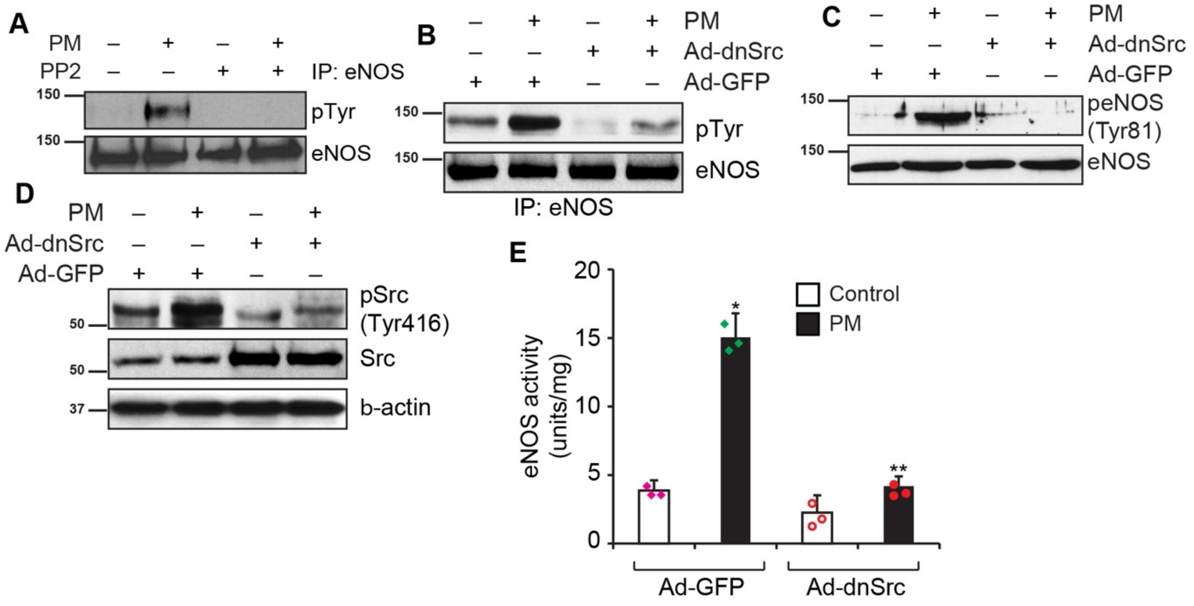

Fig. S6. Src is essential in RIPC-induced eNOS activation. (A) HCAECs were treated with PM for 1 hour in the presence and absence of PP2 (5 μM), and cell lysates were prepared and analyzed for eNOS tyrosine phosphorylation. **(B-E)** HCAECs were transduced with Ad-GFP or Ad-dnSrc and treated with PM for 1 hr., and cell lysates were collected and analyzed for eNOS total tyrosine phosphorylation (B) or eNOS-Tyr81 phosphorylation (C) or analyzed for Src levels and its phosphorylation at Tyr 416 (D) or eNOS activity was assayed and plotted as a bar graph (E). * P < 0.01 versus Ad-GFP control, ** *P* < 0.01 versus Ad-GFP plus PM. One way analysis of variance (ANOVA) was performed using GraphPad -Prism software (version 8)

Fig S7

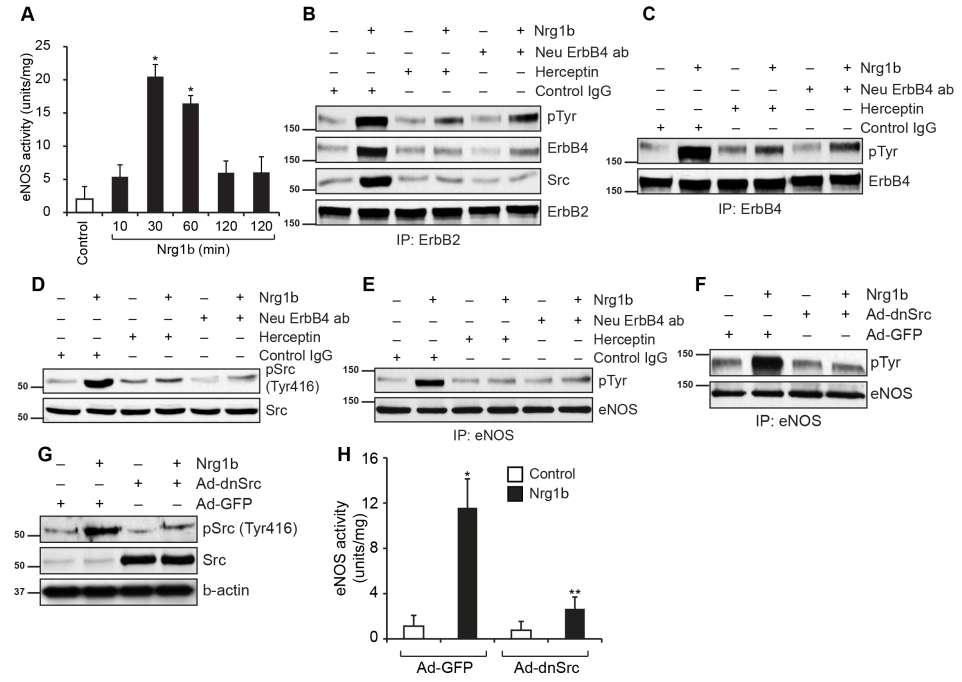

Fig. S7. Nrg1β activates ErbB2/ErbB4-Src-eNOS signaling axis in HCAEC. a, HCAECs were treated with Nrg1β (10 ng/mL) for the indicated period, lysed, and cell lysates were collected. An equal amount of protein from cell lysates was analyzed for eNOS activity and plotted as a bar graph. **b-e,** HCAEC pretreated with Herceptin or neutralizing anti-ErbB4 antibodies and incubated with Nrg1β (10 ng/mL) for 30 min, and cell lysates were prepared. An equal amount of protein from cell lysates was either immunoprecipitated using anti-ErbB2 antibody, and the immunoprecipitates were analyzed by Western blotting using anti-pTyr, anti-ErbB2, anti-ErbB4 and anti-Src antibodies **(b)** or immunoprecipitated with anti-ErbB4 antibodies and analyzed for ErbB4 tyrosine phosphorylation **(c)** or analyzed for Src activation by Western blotting using anti-phospho-Src (Tyr416) antibodies **(d)** or eNOS tyrosine phosphorylation **(e)**. **f-h,** HCAECs were transduced with Ad-GFP or Ad-dnSrc and treated with for Nrg1β (10 ng/mL) for 30 min and cell lysates were collected and analyzed for eNOS tyrosine phosphorylation **(f)** or analyzed by Western blotting using anti-phospho-Src (Tyr416), anti-Src and anti-β-actin antibodies **(g)** or assayed for eNOS activity **(h)**. * $P < 0.01$ versus control Ad-GFP, ** $P < 0.01$ Ad-GFP + Nrg1β. One way analysis of variance (ANOVA) was performed using GraphPad -Prism software (version 8).

Fig S8

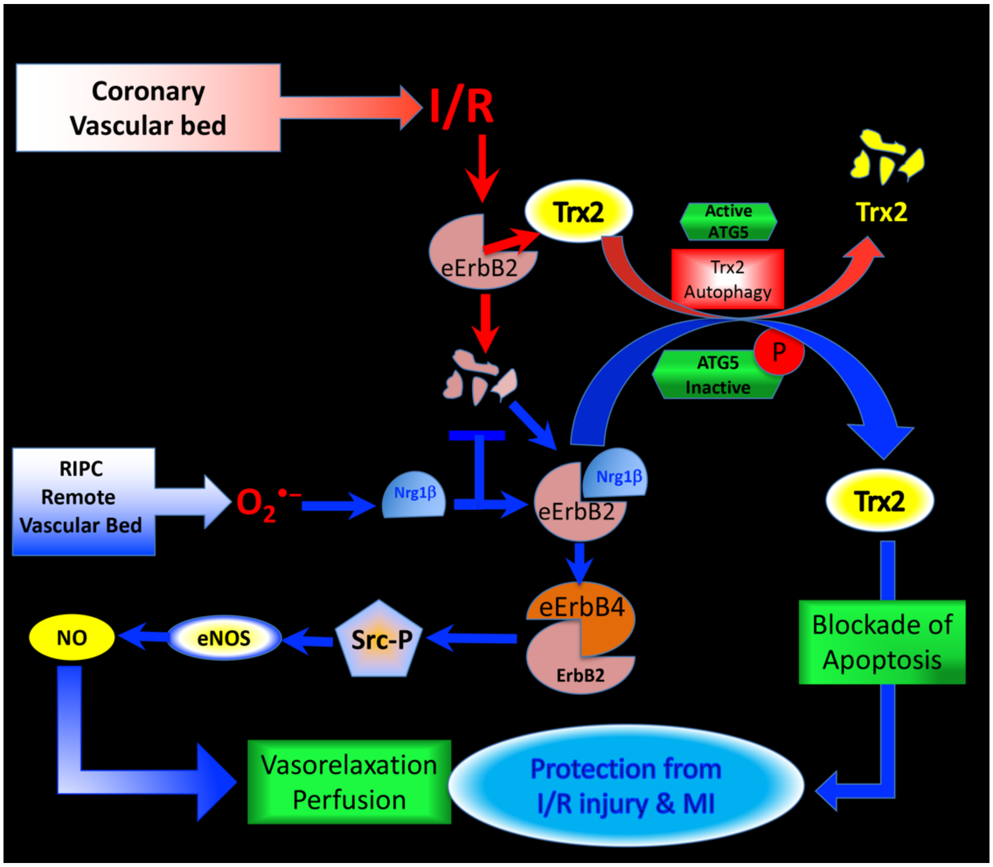

Fig S8. RIPC simultaneously protects against I/R induced impairment of myocardial perfusion and increased myocardial apoptosis due to degradation of Trx2: I/R results in degradation of ErbB2 which causes decreased NO production due to loss of Src-mediated activation of eNOS. ErbB2 degradation also causes ATG5 dephosphorylation resulting in autophagy of Trx2 via activation of ATG5. Simultaneous activation of both of these degradation pathways in I/R causes poor myocardial perfusion and myocardial apoptosis leading to endothelial dysfunction and myocardial apoptosis. RIPC generates superoxide anion (O2.-) that induces the expression and secretion of Nrg1β in a remote vascular bed such as femoral artery. RIPC-mediated Nrg1β prevents ErbB2 degradation by remining bound to ErbB2. Intact ErbB2 is available to interacts with ErbB4 that activates its kinase activity. ErbB2/ErbB4 phosphorylate Src and ATG5 resulting in Src-eNOS mediated NO generation, and inactivation of AT5 due to phosphorylation that prevents autophagy of Trx2. Increase NO facilitate vasorelaxation with better myocardial perfusion. Additionally, rescue of Trx2 prevents myocardial apoptosis resulting in decreased myocardial necrosis and infarction. Both of these pathways converse to prevent I/R mediated myocardial injury and afford protection against I/R injury.
