# Supplementary figures and images for: Nrg1β Released in Remote Ischemic Preconditioning Improves Myocardial Perfusion and Decreases Ischemia/Reperfusion Injury via ErbB2-Mediated Rescue of Endothelial Nitric Oxide Synthase and Abrogation of Trx2 Autophagy
Source: Arterioscler Thromb Vasc Biol. 2021 May 27;41(8):2293–314. doi: 10.1161/ATVBAHA.121.315957 (PMC8288485; doi:10.1161/ATVBAHA.121.315957)

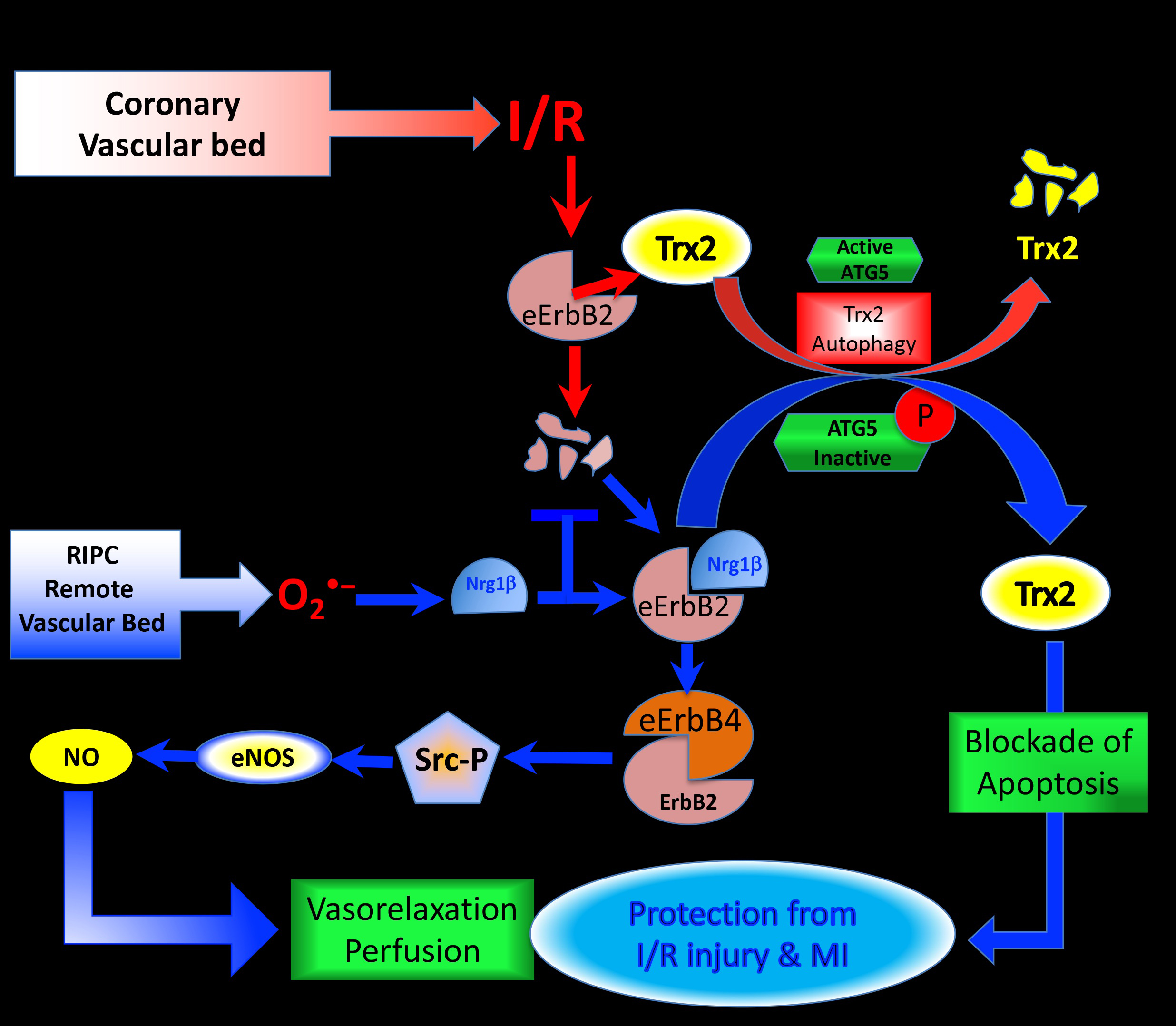

Supplement: Supplementary file 2 [file atv-41-2293-s002.jpg]
